# Transcultural adaptation, validity, and reliability of the SINBAD System Classification for Brazilian Portuguese*

**DOI:** 10.1590/1518-8345.7768.4722

**Published:** 2025-11-10

**Authors:** Julliany Lopes Dias, Eline Lima Borges, Danubia Mendes e Torres, Mônica Antar Gamba, Ângela Lima Pereira, Suelen Gomes Malaquias, Luciana da Silva Barros, Marlene Andrade Martins, Rafael Alves Guimarães, Maria Márcia Bachion

**Affiliations:** 1 Universidade Federal de Goiás, Faculdade de Enfermagem, Goiânia, GO, Brazil. Universidade Federal de Goiás Faculdade de Enfermagem GO Goiânia Brazil; 2 Universidade Federal do Tocantins, Palmas, TO, Brazil. Universidade Federal do Tocantins TO Palmas Brazil; 3 Universidade Federal de Minas Gerais, Escola de Enfermagem, Belo Horizonte, MG, Brazil. Universidade Federal de Minas Gerais Escola de Enfermagem MG Belo Horizonte Brazil; 4 Prefeitura Municipal de Belo Horizonte, Unidade de Referência em Saúde Padre Eustáquio, Belo Horizonte, MG, Brazil. Prefeitura Municipal de Belo Horizonte Unidade de Referência em Saúde Padre Eustáquio MG Belo Horizonte Brazil; 5 Universidade Federal de São Paulo, Escola Paulista de Enfermagem, Departamento de Saúde Coletiva, São Paulo, SP, Brazil. Universidade Federal de São Paulo Escola Paulista de Enfermagem Departamento de Saúde Coletiva SP São Paulo Brazil; 6 Hospital do Coração Anis Rassi, Clínica Médica e Cirúrgica, Goiânia, GO, Brazil. Hospital do Coração Anis Rassi Clínica Médica e Cirúrgica GO Goiânia Brazil; 7 Universidade Federal de Jataí, Jataí, GO, Brazil. Universidade Federal de Jataí GO Jataí Brazil; 8 Scholarship holder at the Conselho Nacional de Desenvolvimento Científico e Tecnológico (CNPq), Brazil. Scholarship holder at the Conselho Nacional de Desenvolvimento Científico e Tecnológico Brazil

**Keywords:** Translating, Validation Study, Foot Ulcer, Diabetic Foot, Nursing, Brazil

## Abstract

to perform the cross-cultural adaptation of the SINBAD System Classification for Brazilian Portuguese and analyze its content validity and reliability.

clinimetric study, including cross-cultural adaptation of the SINBAD System Classification, used to assess foot ulcers in people with diabetes. Verification of semantic and cultural equivalence and clinical usability using the inter-rater reliability (IRR) index (≥ 0.80); content validity using the Content Validity Ratio (CVR) p < 0.05, and internal consistency using McDonald’s omega (> 0.70). The sample involved nurses, physicians, and people with diabetes-related foot ulcers. Data collection was performed using online forms and in-person clinical evaluation of patients treated at referral services in Goiânia, Jataí, São Paulo, Belo Horizonte, and primary care center in Palmas

fifteen nurses participated in the cognitive debriefing; 20 experts (physicians and nurses) participated in content validation; and 113 patients with 120 diabetes-related foot ulcers participated. Eight items (terms and expressions) from the SINBAD System underwent cross-cultural adaptation. CVR = 0.84 and McDonald’s omega = 0.635 were obtained.

the SINBAD system is adapted to Brazilian Portuguese and has satisfactory content validity indices, but it needs investment to achieve greater robustness in terms of reliability and internal consistency.

## Highlights

**(1)** The SINBAD System is recommended for the assessment of diabetes-related foot ulcers. **(2)** The adaptation of the SINBAD System to Brazilian Portuguese was successful in this study. **(3)** The SINBAD System adapted for Brazil achieved good evidence of content validity. **(4)** The internal consistency of the SINBAD System Classification was estimated for the first time. **(5)** The analysis of the internal consistency of the SINBAD System indicates the need for further studies.

## Introduction

Diabetes-related foot ulcers, formerly known as diabetic foot ulcers^(1)^, affect approximately 26 million people worldwide each year^(2)^; they account for about 20% of hospital admissions and are the leading cause of non-traumatic amputations^(3)^.

Prevention through educational interventions for self-care, periodic foot assessments with ulceration risk stratification, and the implementation of evidence-based strategies for risk factor management are essential to reduce the incidence of these ulcers and prevent amputations^(4)^.

Once the ulcer has developed, it is necessary to establish effective treatment actions, monitor its evolution, and detect complications early. In this context, the use of systems for classifying diabetes-related foot ulcers is of great importance^(4-5)^. These systems should be integrated into the overall clinical assessment of the patient as a strategy for monitoring healing, risk of amputation, and clinical management, in addition to being useful in epidemiological and clinical study protocols in this area.

There are a variety of systems for assessing the healing of diabetes-related foot ulcers, including the Meggitt-Wagner System^(6)^; the University of Texas (UT) Score^(7)^; the S(AD)SAD System - Size (Area and Depth), infection (Sepsis), ischemia (Arteriopathy), and denervation (Neuropathy)^(8)^; Foster and Edmonds^(9)^; Van Acker/Peters^(10)^; Margolis^(11)^; the PEDIS System - Perfusion, Extent, Depth, Infection, Sensation^(12)^; DEPA System - depth of the ulcer, extent of bacterial colonization, phase of ulcer healing and the associated underlying etiology^(13)^; CHS System - Curative Health Services^(14)^; DUSS System - Diabetic Ulcer Severity Score^(15)^; SINBAD Classification System - Site, Ischemia, Neuropathy, Bacterial Infection, Area and Depth^(16)^; MAID - Multiple, Ankle Pulse absent, Infection, Duration^(17)^; SEWSS - Saint Elian Wound Score System^(18)^; WifI - Wound, Ischemia, and Infection^(19)^ and DIAFORA - Diabetic Foot Risk Assessment^(20)^.

Nevertheless, depending on the context or purpose of use, all these systems have limitations. There is no gold standard, and the choice is made according to the objective of the professional and/or scientific research. Most systems focus only on the local pathology of diabetes-related foot ulcers and do not adequately assess all parameters relevant to ulcer healing^(5)^.

In Brazil, the Ministry of Health adopts the University of Texas (UT) score and makes it available in a translated version, without details on the translation and cross-cultural adaptation process^(21)^. The Wagner, S(AD)SAD, and UT systems were used in a comparative study conducted in Brazil^(22)^, which also does not mention adaptation or validation.

The consensus of the Brazilian Society of Angiology and Vascular Surgery^(23)^ and the translated version of the International Working Group on the Diabetic Foot (IWGDF) guideline^(24)^ present simplified translations of international classifications, such as WIfI and SINBAD, respectively. However, they did not undergo a formal process of translation and cross-cultural adaptation, which may compromise the accuracy and applicability of the systems in the Brazilian context.

Although WIfI and SINBAD are recommended by the IWGDF^(24)^ for the assessment of diabetes-related foot ulcers, SINBAD stands out for being a concise instrument and for including the assessment of neuropathy, an aspect not covered by WIfI. This feature makes SINBAD particularly useful for communication between health professionals, screening of more severe cases, and referral to specialized teams^(5)^.

In Brazil, care for people with diabetes-related foot ulcers is provided by the Unified Health System (SUS) at all levels of care, among which Primary Health Care stands out in the prevention, identification of complications, and necessary referrals to referral services^(21)^. In this process, the use of assessment tools for screening, monitoring progress, clinical decision-making, and appropriate referral is essential.

The SINBAD system is a simplification of S(AD)SAD^(8)^ and has six assessment elements: Site, Ischemia, Neuropathy, Bacterial Infection, Area, and Depth, forming the acronym SINBAD^(16)^. The evaluation of these items results in a binary score (0 or 1) and a maximum score of six points, which makes this system easy to understand and apply in clinical practice.

The SINBAD System Classification^(16)^ is considered one of the simplest and most complete systems^(24)^, with studies conducted abroad demonstrating good interobserver reproducibility^(25-26)^. In addition, SINBAD has been associated with predictive factors for amputation, reinforcing its clinical utility^(16,27-28)^.

However, to date, there are no records of its cross-cultural adaptation to other languages, including Brazilian Portuguese. In addition, no studies have been identified that investigate its internal consistency^(29)^ or content validity, which are fundamental aspects for ensuring the applicability and accuracy of the instrument in different cultural and linguistic contexts. The absence of such evidence limits its use in populations that do not speak the original language of the instrument, highlighting the need for studies that address these aspects to expand its global applicability.

This study is justified by the need for a system with evidence of validity and reliability to assess diabetes-related foot ulcers, appropriate to the Brazilian reality, which includes factors related to the patient, the ulcer, and complications, is easy to understand and apply, and can be used in clinical practice, allowing accurate decision-making by professionals who care for people with diabetes, especially in primary health care. Thus, the objective of this study is to perform the cross-cultural adaptation of the SINBAD System Classification into Brazilian Portuguese and analyze its content validity and reliability.

## Method

### Study design

A multicenter clinimetric study^(30)^ developed with a focus on cross-cultural adaptation, content validation, and reliability in relation to the internal consistency requirement of the SINBAD System Classification^(16)^.

### Context

The study was conducted between October 2022 and July 2024. The study sites included the virtual environment for the cross-cultural adaptation and content validation stages.

For internal consistency, the scenarios were five referral outpatient services for the care of people with diabetes-related foot ulcers, two in Goiânia-GO, one in Jataí-GO, one in São Paulo-SP, and one in Belo Horizonte-MG, in addition to ten primary care center in Palmas-TO.

Due to the multiplicity of stages and procedures involved, for a better understanding of the design, information such as population, sample, inclusion and exclusion criteria, sources, data collection, and organization adopted in each of them will be presented in a grouped manner.

### Cross-cultural adaptation

The cross-cultural adaptation of the SINBAD System Classification was based on international guidelines and involved six stages: initial translation, synthesis of translations, back-translation, review by a committee of experts, and cognitive debriefing^(31-33)^. It took place from October 2022 to June 2023, with prior authorization from the corresponding author of the original publication.

Initially, the 23 components of the system (terms/sentences) were translated from English into Brazilian Portuguese by a specialized company, which followed criteria for selecting translators in accordance with the reference guidelines^(31-33)^. Thus, two independent bilingual translators (English-Portuguese) were appointed, whose mother tongue was Portuguese, one of whom was familiar with technical terminology in the field and the other without mastery of the subject matter. In summarizing the translations, the translators and committee of experts reached a consensus on the differences and discussed the adoption of terms appropriate to the context of the field and Brazil. The committee of experts included five doctors, nurses with expertise in diabetes-related foot ulcers, a language professional, and the two translators involved. Interaction with the corresponding author of the original study took place via email and virtual meetings. The committee of experts reviewed the translations and developed the version of the instrument, which underwent a back-translation process carried out by two native English translators who were blind to the original version, resulting in the version to be submitted to the next stage of adaptation.

To assess the comprehension and equivalence of the translation in terms of Brazilian culture and identify possible problems in relation to its use in clinical practice, a cognitive debriefing stage was carried out with nurses, who were selected using the snowball technique, based on the initial invitation by the researchers. To select professionals and include them in the sample as judges in this stage, the essential requirement was to have at least six months of experience in caring for people with diabetes-related foot ulcers.

An online form, structured in Google Forms*®*, made available via email, was used to collect data for this stage. The instrument included items for the professional characterization of the participants (area of graduation, degree, and length of experience) and the translated version of the SINBAD System Classification. The judges evaluated the semantic and cultural comprehensibility and clinical usability of the items using the options: absent, partial, or total. Comments and suggestions sent by participants and items that presented a concordance index below the reference value (≥ 80.0%) were reviewed by the committee of experts. Changes considered relevant by the committee were incorporated into the scale for subsequent submission to the content validity verification process.

### Content validity

To assess content validity, a committee of experts was formed^(35)^, recruited by non-probabilistic, intentional sampling from July to September 2023, using the Lattes Platform (National Council for Scientific and Technological Development - Brazil), combined with the snowball technique^(34)^.

The inclusion criterion for participation as an expert in the assessment of content validity was to achieve a minimum score of five points in the scoring system^(36)^, which considers clinical experience, teaching, research, and publications in the field, in addition to academic qualifications, as detailed below:

- *Clinical experience*: Professionals with at least two years of experience in caring for people with diabetes and foot ulcers – mandatory (4 points).
- *Teaching*: Participation in teaching activities, focusing on the management of diabetes-related foot ulcers, for at least one year (1 point).
- *Academic background*: Professionals with a doctorate in the field of diabetes and foot ulcers (2 points); master’s degree in the same field (1 point). Residencies and specializations in related fields, such as vascular surgery, orthopedics, endocrinology, dermatology, or wound treatment (1 point).

In this stage, the same professional characterization instrument used in the previous phase was used for data collection; and, to assess the relevance or representativeness of the items, a Likert scale containing the following response options was used: 1) completely unsatisfactory; 2) very unsatisfactory; 3) perhaps somewhat satisfactory; 4) satisfactory; 5) very satisfactory. This instrument was adapted from the proposal by Coluci, Alexandre, and Milani^(37)^, structured in Google Forms^®^, and sent by email to 40 professionals considered eligible based on the data available in their respective résumés and 81 nominees through the snowball technique^(34)^.

Confirmation of compliance with the inclusion criteria was obtained by consulting the responses indicated in the professional characterization form.

### Reliability

The sample size to verify internal consistency was estimated at least 100 patients, considering the recommendations for scales composed of less than 10 items^(38)^. The non-probability convenience sampling involved individuals with diabetes-related foot ulcers, treated in the research settings, aged 18 years or older, regardless of the time of diabetes diagnosis or ulcer duration.

For data collection, a brief interview was conducted, guided by a structured script, which included items related to sociodemographic and clinical data of the patients. Next, the SINBAD System Classification was applied to assess the patients’ foot ulcers.

In the case of more than one ulcer in this location, the most severe ulcer was evaluated, based on depth, signs of infection, and area. In cases of ulcers on both feet, each was assessed as a separate occurrence, so a patient could have two lesions assessed, one on each foot, applying the respective forms related to the SINBAD System Classification to each one.

All field researchers participated in a 30-minute face-to-face training session with practical activities and reading and discussion of a guide on how to use the instrument.

Patients were evaluated after cleaning and debridement of the ulcer (when necessary) during routine care. To evaluate the items and score the system, the recommendations of the original instrument were adopted^(16)^.

The *site* item was evaluated by inspection and identification of the anatomical region of the foot where the ulcer was present^(16)^. The ischemia item was evaluated by assessing the arterial blood flow in the leg affected by the ulcer, through palpation of the pulses of the pedal artery, posterior tibial artery, and analysis of the patient’s clinical history^(16)^, in addition to additional tests, such as the provoked ischemia maneuver and capillary refill time.

To assess *neuropathy*, a test was performed with a 5.07 or 10 g nylon monofilament, as recommended in the literature^(16,24)^. For wound infection, clinical signs of infection in soft tissue or bone were considered, according to the guidelines proposed by the Infectious Diseases Society of America and the International Working Group on the Diabetic Foot (IWGDF/IDSA)^(4,16)^ and the International Wound Institute (IWII)^(39)^.

In assessing the area item, the two maximum diameter dimensions were measured at right angles with a disposable, sterile paper ruler and multiplied (length x width) to calculate the ulcer area^(16)^. Depth was assessed by observing the affected tissues through inspection of the wound bed, seeking to visualize the compromised structures, such as the epidermis, dermis, subcutaneous tissue, muscles, tendons, or deeper tissues^(16)^.

### Data processing and analysis

In the descriptive analysis of the participants’ professional, sociodemographic, and clinical data, absolute and relative frequencies were used for qualitative variables. For quantitative variables, measures of central tendency (mean or median) and dispersion (standard deviation - SD or interquartile range - IQ) were calculated, depending on whether the distribution was normal or not. The normality of the quantitative variables was verified by the Kolmogorov-Smirnov test with Lilliefors correction (n>30).

The Concordance Index (CI) between the judges in the cognitive debriefing stage was calculated considering the number of agreements (A), that is, agreements between judges for the total option in the evaluation of the semantic, cultural, and clinical usability of the items, divided by the number of agreements (A) added to the disagreements (D), which indicated the number of judges who participated in the stage, using the following formula:

$$IC=\frac{A}{A+D}\;\left(1\right)$$

Indices equal to or greater than 0.80^(40)^ are acceptable.

To estimate content validity, the CVR (*Content Validity Ratio*) was calculated, whose expected value depends on the number of experts^(41)^. The following formula was used for this purpose:

$$CVR=\frac{n-\frac{N}{2}}{\frac{N}{2}}\;\left(2\right)$$

where n = number of experts who agree that the item is *satisfactory(4) or very satisfactory (5)*; and N = number of experts who evaluated the content.

The value obtained was compared to the critical CVR value for significance of 0.05^(42)^ and considered valid if equal to or greater than the critical CVR.

McDonald’s omega was used to analyze internal consistency, accepting values > 0.70^(43)^.

All research data were entered into Vanderbilt University’s Research Electronic Data Capture (REDCap) platform and exported to Microsoft Excel^®^ version 2408, and from there to IBM SPSS Statistics version 28.0 for descriptive analyses and to the R 4.1.2 program^(44)^ to calculate McDonald’s omega.

### Ethical aspects

This study was approved by the Research Ethics Committee (CAAE: 69265323.0.1001.5078).

## Results

In the cross-cultural adaptation of the SINBAD System Classification, eight of the 23 components (terms/sentences) analyzed were changed to meet idiomatic and cultural understanding and usability in clinical practice.

The first changes occurred in the translation synthesis phase by the expert committee, in which the item *bacterial infection* was adapted to wound infection. In addition, the expert committee proposed adapting the title of the scale, translated as SINBAD Classification System to SINBAD Assessment System, since the system does not allow classification, but rather a score referring to the overall assessment of the ulcer.

There were translation discrepancies regarding the use of different but synonymous terms, which were resolved at a meeting of the expert committee. Terms that best suited the Brazilian clinical context were chosen. An example of this is the terms “score” and “point score,” for which the latter was chosen. Other items were translated identically but needed adaptation, such as intact blood flow in the foot, which was adapted to blood flow without alteration in the foot, and sense of protection, adapted to protective sensitivity.

In the back-translation, a version with the same meaning as the original version of the SINBAD system was obtained. The translated, synthesized, and back-translated material was discussed with the corresponding author, William Jeffcoate, who agreed with the changes, thus arriving at the pre-final version to be submitted to cognitive debriefing.

Fifteen nurses, aged between 25 and 65 years, with between 3 and 40 years of experience in the field, participated as judges in the cognitive debriefing. Most had specialization degrees in dermatology or stoma therapy (n = 7) and master’s degrees (n = 5).

The semantic and cultural comprehensibility and usability in clinical practice of the translated version of the SINBAD system achieved satisfactory levels of agreement among the judges for all items (Table 1), except for the description of one of the options for the ischemia assessment result: *Clinical evidence of reduced blood flow in the foot*, and for the description of one of the options for the neuropathy assessment result: Protective sensitivity intact.

In the *cognitive debriefing*, the judges indicated changes, which were discussed by the committee of experts and then implemented, generating a preliminarily adapted version (Figure 1).

Regarding content validation, of the 121 invitations sent, 23 were returned (response rate of 19.0%). Among the respondents, three were excluded for not achieving a score according to the expert selection criteria adopted in this study. Thus, the sample at this stage consisted of 20 participants (10 nurses and 10 physicians), classified according to the reference adopted in master experts (score between six and 20 points; n = 3) and senior experts (score above 20 points; n = 17), aged between 34 and 70 years, with between 2 and 50 years of work experience in the field.

**Table 1-:** Agreement index among judges regarding semantic and cultural comprehensibility and usability in clinical practice of terms/phrases in the SINBAD system (n = 15). Goiânia-GO, Belo Horizonte-MG, Jataí-GO, Palmas-TO, São Paulo-SP, Brazil, 2024

SINBAD* System Classification terms/sentences	**SC** ^†^	**CC** ^‡^	**UPC** ^§^
SINBAD Assessment System*	0.86	0.86	0.93
Category	1.00	0.93	0.93
Definition	0.93	0.93	0.93
Site	0.8	0.86	0.86
Forefoot	0.93	0.86	0.93
Midfoot and hindfoot	0.93	0.86	0.93
Ischemia	1.00	1.00	1.00
Blood flow unchanged in the foot, with at least one palpable pulse	0.93	0.86	0.86
Clinical evidence of reduced blood flow in the foot	0.80	0.86	0.73
Neuropathy	1.00	1.00	1.00
Protective sensation intact	0.73	0.8	0.73
Protective sensation impaired	0.93	0.93	0.93
Wound infection	0.93	0.93	0.93
Absent	1.00	1.00	1.00
Present	1.00	1.00	1.00
Area	1.00	1.00	1.00
Ulcer < 1 cm²	1.00	1.00	0.93
Ulcer ≥ 1 cm²	1.00	1.00	0.93
Depth	1.00	1.00	1.00
Ulcer affects only skin and subcutaneous tissue	0.93	0.93	0.93
Ulcer affects muscle, tendon, or deeper tissue	0.86	0.93	0.93
Score	1.00	0.93	1.00
Total possible score	1.00	0.93	1.00

*SINBAD = Site, Ischemia, Neuropathy, Bacterial Infection, Area and Depth; ^†^SC = Semantic comprehensibility; ^‡^CC = Cultural comprehensibility; ^§^UPC = Usability in clinical practice

**Figure 1-:** Translated version and preliminary version adapted from the SINBAD System Classification for Brazilian Portuguese. Goiânia-GO, Belo Horizonte-MG, Jataí-GO, Palmas-TO, São Paulo-SP, Brazil, 2024

**Preliminary version - translated**			**Preliminary version – adapted (cognitive debriefing phase)**		
SINBAD* Assessment System*			SINBAD* system for diabetic foot ulcer assessment		
Category	Definition	Score	Assessment item	Definition	Score
Site	Forefoot	0	Site	Forefoot	0
	Midfoot and hindfoot	1		Midfoot and hindfoot	1
Ischemia	Blood flow unchanged in the foot, with at least one palpable pulse	0	Ischemia	Arterial blood flow unchanged in the foot, with at least one palpable pulse	0
	Clinical evidence of reduced blood flow in the foot	1		Clinical evidence of reduced arterial blood flow in the foot	1
Neuropathy	Protective sensation intact	0	Neuropathy	Protective sensation preserved	0
	Protective sensation compromised	1		Protective sensation compromised	1
Wound infection	Absent	0	Wound infection	Absent	0
	Present	1		Present	1
Area	Ulcer < 1 cm²	0	Area	Ulcer < 1 cm²	0
	Ulcer ≥ 1 cm²	1		Ulcer ≥ 1 cm²	1
Depth	Ulcer affects only skin and subcutaneous tissue	0	Depth	Ulcer affects only skin and subcutaneous tissue	0
	Ulcer affects muscle, tendon, or deeper tissue	1		Ulcer affects muscle, tendon, or deeper tissue	1
**Total possible score**		**6**	**Total possible score**		**6**

*SINBAD = Site, Ischemia, Neuropathy, Bacterial Infection, Area and Depth

In the first round, it was found that the SINBAD system adapted to Brazilian Portuguese achieved acceptable content validity (Table 2), but adjustments were necessary to achieve adequate clarity, as noted by the experts, specifically in the title and description of the options for the site and ischemia items. Changes were made to the title and the ischemia item. However, the options for the site item were maintained in order to preserve the content of the original instrument.

Once the suggestions had been implemented, the new version was sent to the experts for analysis in a second round, in which 12 of them responded. With the changes made, adequate CVRs were achieved for all the items in the second round (Table 3).

After content validation, the final version of the SINBAD System Classification for Brazilian Portuguese was produced (Figure 2).

**Table 2-:** Validity of SINBAD* System content for assessing diabetes-related foot ulcers - first round of validation (n = 20). Brazil, 2024

**Items evaluated**	**n**_e_^†^	**CVR** ^‡^	**CVR** ^‡^ **critical**
Title: SINBAD* system for assessing diabetic foot ulcers	15	0.500	0.500
Site	18	0.800	0.500
Ischemia	16	0.600	0.500
Neuropathy	17	0.700	0.500
Wound infection	18	0.800	0.500
Area	18	0.800	0.500
Depth	19	0.900	0.500
Options for assessing Site: (0) forefoot (1) midfoot and hindfoot	15	0.500	0.500
Options for assessing Ischemia: (0) Unaltered arterial blood flow in the foot, with at least one palpable pulse (1) Clinical evidence of reduced arterial blood flow in the foot	13	0.300	0.500
Options for assessing Neuropathy: (0) Preserved protective sensitivity (1) Compromised protective sensitivity	16	0.600	0.500
Options for assessing Wound Infection: (0) Absent (1) Present	17	0.700	0.500
Options for assessing Area: (0) Ulcer < 1 cm² (1) Ulcer ≥ 1 cm²	17	0.700	0.500
Options for assessing Depth: (0) Ulcer only affects skin and subcutaneous tissue (1) Ulcer affects muscle, tendon or deeper tissue	18	0.800	0.500
**Average items**		**0.662**	**0.500**

*SINBAD = Site, Ischemia, Neuropathy, Bacterial Infection, Area and Depth; ^†^n_e_ = Number of experts who considered the item essential; ^‡^CVR = Content Validity Ratio

**Table 3-:** Content validity of the SINBAD* system for assessing diabetes-related foot ulcers - second round of validation (n = 12). Brazil, 2024

**Items evaluated**	**n**_e_^†^	**CVR** ^‡^	**CVR** ^‡^ **critical**
Title: SINBAD* system for assessing diabetes-related foot ulcers	11	0.833	0.667
Site	11	0.833	0.667
Ischemia	11	0.833	0.667
Neuropathy	11	0.833	0.667
Wound infection	11	0.833	0.667
Area	11	0.833	0.667
Depth	11	0.833	0.667
Options for assessing Site: (0) forefoot (1) midfoot and hindfoot	12	1.000	0.667
Options for assessing Ischemia: (0) Foot with at least one palpable pulse and no signs and/or symptoms of ischemia (1) Foot with non-palpable pulses and/or presence of signs or symptoms of ischemia	11	0.833	0.667
Options for assessing Neuropathy: (0) Preserved protective sensitivity (1) Compromised protective sensitivity	11	0.833	0.667
Options for assessing Wound Infection: (0) Absent (1) Present	11	0.833	0.667
Options for assessing Area: (0) Ulcer < 1 cm² (1) Ulcer ≥ 1 cm²	11	0.833	0.667
Options for assessing Depth: (0) Ulcer only affects skin and subcutaneous tissue (1) Ulcer affects muscle, tendon or deeper tissue	11	0.833	0.667
**Overall average of items**	**---**	**0.833**	**0.667**

*SINBAD = Site, Ischemia, Neuropathy, Bacterial Infection, Area and Depth; ^†^n_e_ = Number of experts who considered the item essential; ^‡^CVR = Content Validity Ratio

**Figure 2-:** SINBAD* system for assessing diabetes-related foot ulcers - Version adapted from the SINBAD System Classification for Brazilian Portuguese, 2024

SINBAD* system for assessing diabetes-related foot ulcers		
Evaluation item	Definition	Score
Site	Forefoot	0
	Midfoot and rearfoot	1
Ischemia	Foot with at least one palpable pulse and no signs and/or symptoms of ischemia	0
	Foot with non-palpable pulses and/or signs or symptoms of ischemia	1
Neuropathy	Preserved protective sensitivity	0
	Compromised protective sensitivity	1
Wound infection	Absent	0
	Present	1
Area	Ulcer < 1 cm²	0
	Ulcer ≥ 1 cm²	1
Depth	Ulcer affects only the skin and subcutaneous tissue	0
	Ulcer affects muscle, tendon or deeper tissue	1
**Total possible score**		**6**

*SINBAD = Site, Ischemia, Neuropathy, Bacterial Infection, Area and Depth

For the internal consistency analysis, 113 patients took part, who had 120 diabetes-related foot ulcers. Of these, 29.2% were women and 70.8% were men. Age ranged from 23 to 89 years, with a mean of 61.23 years (SD 12.58). The median time since diagnosis of diabetes was 15 years (IQR: 10-25 years) with a range from 1 to 52 years. The median ulcer area was 2.10 cm² (IQR: 2.0-24 cm²) with a range from 0.10 cm² to 64.60 cm². The median time of injury was 6.50 months (IQR: 2-24 months) with a range of 1 to 365 months. The SINBAD system adapted for Brazilian Portuguese obtained a McDonald’s omega value of 0.635.

## Discussion

This research represents an original contribution since, as far as we know, the cross-cultural adaptation, content validation and internal consistency assessment of the SINBAD System Classification is being carried out for the first time.

The cross-cultural adaptation followed strict methodological standards^(31-33)^, ensuring that the translated version was semantically and culturally equivalent and usable in clinical practice. In this sense, modifications were necessary during the instrument’s adaptation stages, taking into account cultural and regional nuances to ensure that the instrument is understandable.

The modification of the term bacterial infection, originally adopted in the SINBAD System, was adapted to wound infection, as it is more appropriate, since different microorganisms can cause infections^(39)^. This is an important factor to consider when assessing ulcers, since its presence causes a local and/or systemic response in the person, causing local tissue damage and preventing healing^(39)^. Due to the anatomical characteristics of the foot, loss of skin integrity and local infections are particularly important, as they can favor serious processes such as osteomyelitis^(45)^.

The translation of the phrase *intact blood flow in the foot*, modified to *unaltered blood flow in the foot*, is justified since the term intact refers to something that is in perfect condition, without any alterations, which may not be correct. Clinically, the pulse may be present, but with reduced strength and amplitude, so the flow is present, but not necessarily intact. The expression *intact protective sensation* was adapted to *preserved protective sensitivity*, since the term protective sensitivity is what is used in Brazil in the context of semiology^(46)^.

The modification of the scale’s title to *SINBAD Evaluation System* is justifiable, since there is a difference between instruments that only score and those that classify^(47)^. In this context, it is worth remembering that the SINBAD system was originally developed as a scoring system for a global assessment of the ulcer.

Despite this, there have been initiatives to use the scale for classification purposes, as shown in a study in which ulcers were classified as severe (SINBAD score 5-6), moderate risk (SINBAD score 3-4) and low risk (SINBAD score 0-2). It found that patients with severe ulcers had a higher risk of mortality compared to those with low and moderate lesions^(48)^. Another initiative in this direction is that of England and Wales, where the SINBAD system is widely used in national audits. In that context, it has been found that a SINBAD score < 3 results in a 60% ulcer healing rate within 12 weeks, and a major amputation rate within 6 months of 0.7%. For SINBAD scores ≥ 3, these rates are 35% and 2.7% respectively^(49)^. However, these initiatives do not converge with the original proposal, which has no classification^(16)^.

It took two rounds to reach acceptable rates for the content validity of the SINBAD system adapted to Brazilian Portuguese. Although the scale is considered easy and quick to apply, as it only has six assessment items, its use must be unequivocal, which justifies special attention to every detail of the validation process.

The main highlight was the term diabetic foot ulcer, which, despite its consolidated use in the literature and by international organizations such as the International Diabetes Federation (IDF)^(50)^ and the World Health Organization (WHO)^(51)^, had a recommendation for a change of terminology in the IWGDF guidelines, published at the end of 2023^(1)^, adopting diabetes-related foot ulcer, which was widely accepted by the experts, who then recommended the change.

The new terminology is accompanied by a redefinition that broadens the understanding of diabetes-related foot disease^(1)^. Previously, individuals with peripheral neuropathy or peripheral arterial disease without active ulcers could be mistakenly considered not to have foot disease. The new definition^(1)^ recognizes that the presence of these risk factors already characterizes a pathological condition in the feet, even in the absence of visible ulcerations. It is therefore essential to inform patients that if they have peripheral neuropathy or peripheral arterial disease in the lower limbs, they have an ongoing foot disease, requiring preventive interventions to avoid more serious complications, such as the development of ulcers or infections.

In Brazil, it is recommended that primary care should solve 80% of the population’s health problems^(52)^. Thus, the prevention and systematic assessment of diabetes-related foot ulcers are fundamental actions, requiring qualified professionals capable of using appropriate instruments. In this sense, the adapted version of the SINBAD system for assessing diabetes-related foot ulcers could be a very important auxiliary tool.

In terms of internal consistency, McDonald’s omega was lower than 0.7, which suggests that the items in the instrument are not consistently measuring the same construct or factor, which may indicate that the scale is multidimensional. In this sense, it should be noted that the SINBAD System Classification is sometimes mentioned as a tool for assessing diabetes-related foot ulcers^(29)^, as a tool for classifying healing potential^(12)^, and also for lesion severity and amputation prediction^(16)^.

Therefore, carrying out an exploratory factor analysis (EFA) and/or confirmatory factor analysis (CFA) can help verify the factor structure of the instrument and identify whether there are multiple factors influencing the internal consistency value^(46)^.

Despite this weakness, this system has great potential for use throughout the country, especially in the context of primary care, either for auditing, as in England and Wales, as previously mentioned^(49)^, or to justify the referral of affected people to specialized services, as well as serving to monitor events that indicate complications, such as the appearance of infection, in both cases helping to prevent amputation^(27)^.

Although the response rate was low, considering the initial contact for content validation, it is important to note that the profile and number of experts established in the literature was met^(35-36)^.

The availability of a system that has been cross-culturally adapted and tested for content validity can help support the clinical practice of professionals who treat people with diabetes-related foot ulcers. This system makes it possible to track complications, systematize follow-up and refer people to specialized services, giving greater security to the conduct aimed at this population. The tool can help improve the care and monitoring of diabetes-related foot ulcers in Brazil, facilitating communication between health professionals and evidence-based clinical decision-making.

## Conclusion

The SINBAD System Classification has been properly adapted and validated for Brazilian Portuguese, with high levels of agreement between the experts regarding the relevance of the items. This version is ready to be used in clinical practice and is a valid tool for assessing diabetes-related foot ulcers. However, it is important to note that the system still has low internal consistency, which makes it necessary to study the construct validity of the instrument, and possible insertion of new items.

## Data Availability

All data generated or analysed during this study are included in this published article.
