# Supplementary material for: Transcultural adaptation, validity, and reliability of the SINBAD System Classification for Brazilian Portuguese
Source: Rev Lat Am Enfermagem. 2025 Nov 10;33:e4722. doi: 10.1590/1518-8345.7768.4722 (PMC12610932; doi:10.1590/1518-8345.7768.4722)
Supplement: Translation

# Translation: Adaptação transcultural, validade e confiabilidade do SINBAD *System Classification* para o português do Brasil*

**DOI:** 10.1590/1518-8345.7768.4723

**Authors:** Julliany Lopes Dias, Eline Lima Borges, Danubia Mendes e Torres, Mônica Antar Gamba, Ângela Lima Pereira, Suelen Gomes Malaquias, Luciana da Silva Barros, Marlene Andrade Martins, Rafael Alves Guimarães, Maria Márcia Bachion

**Affiliations:** 1 Universidade Federal de Goiás, Faculdade de Enfermagem, Goiânia, GO, Brasil. Universidade Federal de Goiás Faculdade de Enfermagem GO Goiânia Brasil; 2 Universidade Federal do Tocantins, Palmas, TO, Brasil. Universidade Federal do Tocantins TO Palmas Brasil; 3 Universidade Federal de Minas Gerais, Escola de Enfermagem, Belo Horizonte, MG, Brasil. Universidade Federal de Minas Gerais Escola de Enfermagem MG Belo Horizonte Brasil; 4 Prefeitura Municipal de Belo Horizonte, Unidade de Referência em Saúde Padre Eustáquio, Belo Horizonte, MG, Brasil. Prefeitura Municipal de Belo Horizonte Unidade de Referência em Saúde Padre Eustáquio MG Belo Horizonte Brasil; 5 Universidade Federal de São Paulo, Escola Paulista de Enfermagem, Departamento de Saúde Coletiva, São Paulo, SP, Brasil. Universidade Federal de São Paulo Escola Paulista de Enfermagem Departamento de Saúde Coletiva SP São Paulo Brasil; 6 Hospital do Coração Anis Rassi, Clínica Médica e Cirúrgica, Goiânia, GO, Brasil. Hospital do Coração Anis Rassi Clínica Médica e Cirúrgica GO Goiânia Brasil; 7 Universidade Federal de Jataí, Jataí, GO, Brasil. Universidade Federal de Jataí GO Jataí Brasil; 8 Bolsista do Conselho Nacional de Desenvolvimento Científico e Tecnológico (CNPq), Brasil. Bolsista do Conselho Nacional de Desenvolvimento Científico e Tecnológico Brasil

**Keywords:** Tradução, Estudo de Validação, Úlcera do Pé, Pé Diabético, Enfermagem, Brasil

## Abstract

realizar a adaptação transcultural do SINBAD *System Classification* para o português do Brasil e analisar sua validade de conteúdo e confiabilidade.

estudo clinimétrico, incluindo adaptação transcultural do sistema SINBAD *System Classification*, utilizado para avaliar úlceras nos pés de pessoas com diabetes. Verificação de equivalência semântica, cultural e usabilidade clínica mediante índice de concordância (IC) entre avaliadores ( ≥ 0,80); validade de conteúdo por meio do *Content Validity Ratio* (CVR) (*p* < 0,05), e consistência interna pelo ômega de McDonald (> 0,70). A amostra envolveu enfermeiros, médicos e pessoas com úlcera no pé relacionada ao diabetes. Na coleta de dados aplicaram-se formulários *online* e avaliação clínica presencial de pacientes atendidos em serviços de referência em Goiânia, Jataí, São Paulo, Belo Horizonte, e unidades básicas de saúde em Palmas.

participaram 15 enfermeiros no *cognitive debriefing*; 20 *experts* (médicos e enfermeiros) na validação de conteúdo, e 113 pacientes com 120 úlceras no pé relacionadas ao diabetes. Oito itens (termos e expressões) do SINBAD *System* sofreram adaptação transcultural. Obteve-se CVR = 0,84 e ômega de McDonald = 0,635.

o sistema SINBAD está adaptado para o português do Brasil, apresenta índices satisfatórios de validade de conteúdo, contudo necessita de investimentos para alcançar mais robustez quanto à confiabilidade, no quesito consistência interna.

## Destaques

**(1)** O SINBAD *System* é recomendado para avaliação de úlcera no pé relacionada ao diabetes. **(2)** A adaptação do SINBAD *System* para o português do Brasil foi bem-sucedida nesse estudo. **(3)** SINBAD *System* adaptado para o Brasil alcançou boas evidências de validade de conteúdo. **(4)** Pela primeira vez foi estimada a consistência interna do SINBAD *System Classification*. **(5)** A análise da consistência interna do SINBAD *System* indica necessidade novos estudos.

## Introdução

Úlceras nos pés relacionadas ao diabetes, anteriormente designadas como úlceras do pé diabético^(1)^, afetam aproximadamente 26 milhões de pessoas no mundo a cada ano^(2)^; representam cerca de 20% das internações e são a principal causa de amputações não traumáticas^(3)^.

A prevenção, por meio de intervenções educativas para o autocuidado, avaliações periódicas dos pés com estratificação de risco de ulceração e implementação de estratégias baseadas em evidências para a gestão de fatores de risco são indispensáveis para reduzir a incidência das úlceras e prevenir amputações^(4)^.

Uma vez instalada a úlcera, é necessário estabelecer ações efetivas de tratamento, acompanhar a evolução e detectar precocemente complicações. Nesse contexto, a utilização de sistemas para classificação de úlcera no pé relacionada ao diabetes é de grande importância^(4-5)^. Os sistemas devem ser integrados na avaliação clínica global do paciente, como estratégia de acompanhamento da cicatrização, risco de amputação e manejo clínico, além de serem úteis em protocolos de estudos epidemiológicos e clínicos na área.

Há uma variedade de sistemas para avaliar a cicatrização de úlceras nos pés relacionadas ao diabetes, incluindo o Sistema Meggitt-Wagner^(6)^; Pontuação Universidade do Texas (UT)^(7)^; Sistema S(AD)SAD - *Size (Area and Depth), infection (Sepsis), ischemia (Arteriopathy), and denervation (Neuropathy)*^(8)^; Foster e Edmonds^(9)^; Van Acker/Peters^(10)^; Margolis^(11)^; Sistema PEDIS - *Perfusion, Extent, Depth, Infection, Sensation*^(12)^; Sistema DEPA - *depth of the ulcer, extent of bacterial colonization, phase of ulcer healing and the associated underlying etiology*^(13)^; Sistema CHS - *Curative Health Services*^(14)^; Sistema DUSS - *Diabetic Ulcer Severity Score*^(15)^; Sistema de Classificação SINBAD - *Site, Ischemia, Neuropathy, Bacterial Infection, Area and Depth*^(16)^; MAID - *Multiple, Ankle Pulse absent, Infection, Duration*^(17)^; SEWSS - *Saint Elian Wound Score System*^(18)^; WifI - *Wound, Ischemia, and Infection*^(19)^ e DIAFORA - *Diabetic Foot Risk Assessment*^(20)^.

Apesar disso, dependendo do contexto ou propósito de uso, todos esses sistemas têm limitações. Não há um padrão ouro, sendo a escolha realizada de acordo com o objetivo do profissional e/ou da pesquisa científica. A maioria dos sistemas concentra-se apenas na patologia local das úlceras no pé relacionadas ao diabetes e não avaliam adequadamente todos os parâmetros relevantes para a cicatrização da úlcera^(5)^.

No Brasil, o Ministério da Saúde adota a pontuação da Universidade do Texas (UT) e a disponibiliza em uma versão traduzida, sem detalhamento sobre o processo de tradução e adaptação transcultural^(21)^. Os sistemas de Wagner, S(AD)SAD e UT foram utilizados em estudo comparativo realizado no Brasil^(22)^, nele também não é mencionada adaptação ou validação.

O consenso da Sociedade Brasileira de Angiologia e de Cirurgia Vascular^(23)^ e a versão traduzida do *guideline* do *International Working Group on the Diabetic Foot* (IWGDF)^(24)^ apresentam traduções simplificadas de classificações internacionais, como o WIfI e SINBAD, respectivamente. Contudo, não há um processo formal de tradução e adaptação transcultural, o que pode comprometer a precisão e a aplicabilidade dos sistemas no contexto brasileiro.

Embora o WIfI e o SINBAD sejam recomendados pelo IWGDF^(24)^ para avaliação de úlceras nos pés relacionadas ao diabetes, o SINBAD destaca-se por ser um instrumento conciso e por incluir a avaliação da neuropatia, aspecto não contemplado pelo WIfI. Essa característica torna o SINBAD particularmente útil para a comunicação entre profissionais de saúde, triagem de casos mais graves e encaminhamento a equipes especializadas^(5)^.

No Brasil, o atendimento às pessoas com úlcera no pé relacionada ao diabetes é assegurado pelo Sistema Único de Saúde (SUS) em todos os níveis de atenção, entre os quais se destaca a Atenção Primária à Saúde na prevenção, identificação de complicações e encaminhamentos necessários a serviços de referência^(21)^. Nesse processo, é essencial o uso de instrumentos de avaliação para triagem, monitoramento da evolução, tomada de decisões clínicas e encaminhamento adequado.

O sistema SINBAD é uma simplificação do S(AD)SAD^(8)^, e possui seis elementos de avaliação: *Site, Ischemia, Neuropathy, Bacterial Infection, Area* e *Depth,* formando o acrônimo SINBAD^(16)^. A avaliação dos seus itens resulta em uma pontuação binária (0 ou 1) e um escore máximo de seis pontos, o que torna o sistema de fácil compreensão e aplicação na prática clínica^(5)^.

O SINBAD *System Classification*^(16)^ é considerado um dos sistemas mais simples e completos^(24)^, com estudos conduzidos no exterior que demonstram uma boa reprodutibilidade interobservadores^(25-26)^. Além disso, o SINBAD tem sido associado a fatores preditivos de amputação, reforçando sua utilidade clínica^(16,27-28)^.

Porém, até o momento, não há registros de sua adaptação transcultural para outros idiomas, incluindo o português do Brasil. Adicionalmente, não foram identificados estudos que investiguem sua consistência interna^(29)^ ou validade de conteúdo, aspectos fundamentais para garantir a aplicabilidade e a precisão do instrumento em diferentes contextos culturais e linguísticos. A ausência dessas evidências limita sua utilização em populações que não falam o idioma original do instrumento, destacando a necessidade de estudos que abordem esses aspectos para ampliar sua aplicabilidade global.

A realização deste estudo justifica-se pela necessidade de se dispor um sistema adequado à realidade brasileira, com evidências de sua validade e confiabilidade para avaliar a úlcera no pé relacionada ao diabetes, que inclua fatores ligados ao paciente, à úlcera e às complicações, seja de fácil compreensão e aplicação, e que possa ser usado na prática clínica, permitindo tomadas de decisões precisas pelos profissionais que cuidam de pessoas com diabetes, especialmente na atenção primária à saúde. Assim, o objetivo deste estudo é realizar a adaptação transcultural do SINBAD *System Classification* para o português do Brasil e analisar sua validade de conteúdo e confiabilidade.

## Método

### Delineamento

Estudo clinimétrico^(30)^, multicêntrico, desenvolvido com foco na adaptação transcultural, validação de conteúdo e confiabilidade em relação ao requisito de consistência interna do SINBAD *System Classification*^(16)^.

### Contexto

O estudo foi realizado entre outubro de 2022 a julho de 2024. Os locais do estudo incluíram o ambiente virtual, para as etapas de adaptação transcultural e de validação de conteúdo.

Para a consistência interna os cenários foram cinco serviços ambulatoriais de referência para atendimento a pessoas com úlcera no pé relacionada ao diabetes, sendo dois em Goiânia - GO, um Jataí - GO, um em São Paulo - SP e um em Belo Horizonte - MG, além de dez unidades básicas de saúde em Palmas -TO.

Devido à multiplicidade de etapas e de procedimentos envolvidos, para melhor compreensão do delineamento, serão apresentadas informações como população, amostra, critérios de inclusão e exclusão, fontes, coleta e organização de dados adotados em cada uma delas de forma agrupada.

### Adaptação transcultural

A adaptação transcultural SINBAD *System Classification* foi baseada em *guidelines* internacionais, e percorreu seis etapas: tradução inicial, síntese das traduções, retrotradução, revisão pelo comitê de especialistas e *cognitive debriefing*^(31-33)^. O processo ocorreu de outubro de 2022 a junho de 2023, com autorização prévia do autor correspondente da publicação original.

Inicialmente, os 23 componentes do sistema (termos/frases) foram traduzidos do inglês para o português do Brasil por uma empresa especializada, que seguiu critérios de escolha de tradutores conforme os *guidelines* de referência^(31-33)^. Assim, foram indicados dois tradutores independentes bilíngues (inglês-português), cuja língua materna foi o português, sendo um dos tradutores ciente de terminologia técnica da área e outro sem domínio da temática. Na síntese das traduções, os tradutores e comitê de especialistas chegaram a um consenso sobre as divergências e discutiram a adoção de termos adequados ao contexto da área e do Brasil. Fizeram parte do comitê de especialistas cinco doutores, enfermeiros com expertise em úlceras nos pés relacionadas ao diabetes, um profissional de idiomas e os dois tradutores envolvidos. A interação com o autor correspondente do estudo original se deu via *e-mails* e reuniões virtuais. O comitê de especialistas revisou as traduções e desenvolveu a versão do instrumento, que passou pelo processo de retrotradução, efetuada por dois tradutores nativos em inglês, cegos quanto à versão original, chegando-se à versão a ser submetida à próxima etapa da adaptação.

Para avaliar a compreensão e a equivalência da tradução a termos da cultura brasileira e identificar possíveis problemas em relação ao uso na prática clínica, foi realizada a etapa de *cognitive debriefing*^(33)^ com enfermeiros, os quais foram selecionados pela técnica de bola de neve^(34)^, a partir do convite inicial pelos pesquisadores. Para seleção de profissionais e inclusão na amostra como juízes, nesta etapa o requisito essencial era apresentar experiência mínima de seis meses no atendimento a pessoas com úlcera no pé relacionada ao diabetes.

Para a coleta de dados utilizou-se um formulário *online,* estruturado no *Google Forms*^®^, disponibilizado via *e-mail*. O instrumento incluiu itens de caracterização profissiográfica dos participantes (área de graduação, titulação e tempo de experiência) e a versão traduzida do SINBAD *System Classification*. Os juízes avaliaram a compreensibilidade semântica, cultural e a usabilidade clínica dos itens, mediante as opções: ausente, parcial ou total. Comentários e sugestões enviadas pelos participantes e itens que apresentaram índice de concordância abaixo do valor de referência (≥ 80,0%) foram revisados pelo comitê de especialistas. Alterações consideradas pertinentes pelo comitê foram incorporadas à escala para posterior submissão ao processo de verificação de validade de conteúdo.

### Validade de conteúdo

Para avaliação da validade de conteúdo, foi composto um comitê de *experts*^(35)^, recrutados por amostragem não probabilística, intencional, de julho a setembro de 2023, utilizando a Plataforma Lattes (Conselho Nacional de Desenvolvimento Científico e Tecnológico - Brasil), somado à técnica de bola de neve^(34)^.

O critério de inclusão para a participação como *expert* na avaliação da validade de conteúdo foi atingir pontuação mínima de cinco pontos no sistema de pontuação^(36)^, que considera experiência clínica, atuação em ensino, pesquisa e publicações na área, além da titulação acadêmica, conforme detalhado a seguir:

- *Experiência clínica*: Profissionais com ao menos dois anos de experiência no atendimento a pessoas com diabetes e úlceras no pé – obrigatório (4 pontos).
- *Ensino*: Participação em atividades de ensino, com foco na área de manejo de úlceras no pé relacionadas ao diabetes, a pelo menos um ano (1 ponto).
- *Formação acadêmica*: Profissionais com doutorado na área de diabetes e úlceras no pé (2 pontos); mestrado na mesma área (1 ponto). Residências e especializações em áreas afins, como cirurgia vascular, ortopedia, endocrinologia, dermatologia, ou tratamento de feridas (1 ponto).

Nessa etapa, para coleta de dados, empregou-se o mesmo instrumento de caracterização profissiográfica da fase anterior, e, para avaliação da pertinência ou representatividade dos itens, uma escala tipo Likert contendo as seguintes opções de resposta: 1) completamente insatisfatória; 2) muito pouco satisfatória; 3) talvez, de algum modo satisfatória; 4) satisfatória; 5) muito satisfatória. O instrumento foi adaptado a partir da proposta de Coluci, Alexandre e Milani^(37)^, estruturado no *Google Forms*^®^ e enviado por *e-mail* para 40 profissionais considerados elegíveis com base nos dados disponíveis nos respectivos currículos e 81 indicados por meio da técnica de bola de neve^(34)^.

A confirmação do atendimento aos critérios de inclusão ocorreu mediante consulta às respostas indicadas no formulário de caracterização profissiográfica.

### Confiabilidade

O tamanho amostral para verificar a consistência interna foi estimado em, no mínimo, 100 pacientes, considerando as recomendações para escalas compostas por menos que 10 itens^(38)^. A amostragem não probabilística por conveniência envolveu indivíduos com úlcera no pé relacionada a diabetes, em atendimento nos cenários de investigação, com idade igual ou maior a 18 anos, independente do tempo de diagnóstico de diabetes ou tempo da úlcera.

Para a coleta de dados, fez-se uma entrevista breve, guiada por um roteiro estruturado, que incluía itens referentes a dados de caracterização sociodemográfica e clínica dos pacientes. A seguir, foi aplicado o SINBAD *System Classification* para avaliação da úlcera no pé dos pacientes.

Em caso de presença de mais de uma úlcera nesse local, adotou-se para a avaliação aquela considerada mais grave, tendo como referência a profundidade, sinais de infecção e a área. Em caso de úlcera em ambos os pés, cada uma delas foi avaliada como uma ocorrência distinta, assim, um paciente poderia ter duas lesões avaliadas, uma em cada pé, aplicando-se para cada uma os respectivos formulários referentes ao SINBAD *System Classification.*

Todos os pesquisadores de campo participaram de treinamento presencial, de 30 minutos, com atividades práticas e leitura e discussão de um guia de uso do instrumento.

Os pacientes foram avaliados após a limpeza e desbridamento da úlcera (quando necessário) durante o atendimento de rotina. Para avaliação dos itens e pontuação do sistema, foram adotadas as recomendações do instrumento original^(16)^.

O item *local* foi avaliado por meio de inspeção e identificação da região anatômica do pé em que a úlcera se apresentava^(16)^. No item *isquemia* avaliou-se o fluxo sanguíneo arterial da perna afetada pela úlcera, mediante palpação dos pulsos da artéria pediosa, tibial posterior, e análise da história clínica do paciente^(16)^, além de testes adicionais, como a manobra de isquemia provocada e o tempo de enchimento capilar.

Para avaliar o item *neuropatia*, foi realizado o teste com o monofilamento de nylon de 5.07 ou 10 g, conforme recomendação da literatura^(16,24)^. Quanto ao item *infecção na ferida*, foram considerados os sinais clínicos de infecção, em tecido mole ou osso, conforme as diretrizes propostas pela Sociedade de Doenças Infecciosas da América e pelo Grupo de Trabalho Internacional sobre pé diabético (IWGDF/IDSA)^(4,16)^ e do Instituto Internacional de Feridas (IWII)^(39)^.

Na avaliação do item *área*, as duas dimensões máximas de diâmetro foram mensuradas em ângulos retos, com uma régua de papel, descartável e estéril, e multiplicadas (comprimento x largura) para calcular da área da úlcera^(16)^. A *profundidade* foi avaliada pela observação dos tecidos afetados, mediante a inspeção do leito da ferida, buscando-se visualizar as estruturas comprometidas, tais como epiderme, derme, tecido subcutâneo, músculos, tendões ou tecidos mais profundos^(16)^.

### Tratamento e análise dos dados

Na análise descritiva dos dados profissiográficos, sociodemográficos e clínicos dos participantes, foram utilizadas, para as variáveis qualitativas, as frequências absolutas e relativas. Para as variáveis quantitativas, foram calculadas medidas de tendência central (média ou mediana) e dispersão (Desvio-Padrão - DP ou Intervalo Interquartílico – IIQ), conforme a normalidade ou não da distribuição. A normalidade das variáveis quantitativas foi verificada pelo teste Kolmogorov-Smirnov com correção de Lilliefors (n > 30).

O Índice de Concordância (IC) entre os juízes na etapa de *cognitive debriefing* foi calculado considerando o número de acordos (A) ou seja, concordâncias entre os juízes para a opção *total* na avaliação da compreensibilidade semântica, cultural e usabilidade clínica dos itens, dividido pelo número de acordos (A) somados aos desacordos (D), que indicavam o número de juízes que participaram da etapa, por meio da seguinte fórmula:

$$IC=\frac{A}{A+D}\;\left(1\right)$$

Sendo aceitáveis índices iguais ou maiores que 0,80^(40)^.

Para estimar a validade de conteúdo foi calculado o CVR (*Content Validity Ratio*), cujo valor esperado depende do número de *experts*^(41)^. Para isso foi utilizada a seguinte fórmula:

$$CVR=\frac{n-\frac{N}{2}}{\frac{N}{2}}\;\left(2\right)$$

onde, *n* = número de *experts* que concordam que o item é *satisfatório (4)* ou *muito satisfatório (5)*; e N = número de *experts* que avaliaram o conteúdo.

O valor obtido foi comparado ao valor de CVR crítico para significância de 0,05^(42)^ e considerado válido o valor igual ou maior ao CVR crítico.

Para análise da consistência interna foi utilizado o ômega de McDonald, aceitando-se valores > 0,70^(43)^.

Todos os dados da pesquisa foram inseridos na plataforma *Research Eletronic Data Capture* (REDCap) da *Vanderbilt University*, e exportados para o *softwareMicrosoft Excel*^®^ versão 2408, e deste para o IBM SPSS *Statistics* versão 28.0 para as análises descritivas e para o programa R 4.1.2^(44)^, para calcular o ômega de McDonald.

### Aspectos éticos

Este estudo foi aprovado pelo Comitê de Ética em Pesquisa (CAAE: 69265323.0.1001.5078).

## Resultados

Na adaptação transcultural do SINBAD *System Classification,* oito dos 23 componentes (termos/frases) analisados sofreram alteração, para atender à compreensão idiomática, cultural e usabilidade na prática clínica.

As primeiras alterações ocorreram na fase de síntese das traduções, pelo comitê de especialistas, em que o item *bacterial infection* traduzido como *infecção bacteriana* foi adaptado para *infecção na ferida*. Além disso, o comitê de especialistas propôs a adaptação do título da escala, traduzido como Sistema de Classificação SINBAD para Sistema de Avaliação SINBAD, uma vez que o sistema não permite a classificação, mas sim uma pontuação referente à avaliação global da úlcera.

Ocorreram divergências de tradução relativos ao uso de termos diferentes, porém sinônimos, e foram resolvidas em reunião do comitê de especialistas. Foram escolhidos termos que melhor atendiam o contexto clínico brasileiro. Como exemplo citam-se os termos *escore* e *pontuação,* para os quais optou-se pelo segundo. Outros itens foram traduzidos de forma idêntica, mas precisaram de adaptação, tais como: *fluxo sanguíneo intacto no pé* que foi adaptado para *fluxo sanguíneo sem alteração no pé*, e *sensação de proteção,* adaptado para *sensibilidade protetora*.

Na retrotradução, foi obtida uma versão com o mesmo sentido da versão original do sistema SINBAD. O material traduzido, sintetizado e retrotraduzido foi discutido com o autor correspondente, William Jeffcoate, que concordou com as alterações, chegando-se, assim, à versão pré-final, a ser submetida ao *cognitive debriefing.*

Participaram como juízes no *cognitive debriefing* 15 enfermeiros, com faixa etária entre 25 e 65 anos, tempo de atuação na área entre 3 e 40 anos. A maioria apresentava titulação de especialização na área de dermatologia ou estomaterapia (n = 7), e mestrado (n = 5).

A compreensibilidade semântica, cultural e usabilidade na prática clínica da versão traduzida do sistema SINBAD alcançou índices satisfatórios de concordância entre os juízes para todos os itens (Tabela 1), exceto para a descrição de uma das opções para o resultado da avaliação de isquemia: *Evidência clínica de fluxo sanguíneo reduzido no pé*, e para a descrição de uma das opções para o resultado de avaliação de neuropatia: *Sensibilidade protetora intacta*.

No *cognitive debriefing* os juízes indicaram alterações, as quais foram discutidas pelo comitê de especialistas, e, então, implementadas, gerando-se uma versão preliminarmente adaptada (Figura 1).

**Tabela 1-:** Índice de concordância entre os juízes quanto à compreensibilidade semântica, cultural e usabilidade na prática clínica dos termos/ frases do sistema SINBAD (n = 15). Goiânia-GO, Belo Horizonte-MG, Jataí-GO, Palmas-TO, São Paulo-SP, Brasil, 2024

Termos/frases do SINBAD* *System Classification*	**CS** ^†^	**CC** ^‡^	**UPC** ^§^
Sistema de Avaliação SINBAD*	0,86	0,86	0,93
Categoria	1,00	0,93	0,93
Definição	0,93	0,93	0,93
Local	0,8	0,86	0,86
Antepé	0,93	0,86	0,93
Mediopé e retropé	0,93	0,86	0,93
Isquemia	1,00	1,00	1,00
Fluxo sanguíneo sem alteração no pé, com pelo menos um pulso palpável	0,93	0,86	0,86
Evidência clínica de fluxo sanguíneo reduzido no pé	0,80	0,86	0,73
Neuropatia	1,00	1,00	1,00
Sensibilidade protetora intacta	0,73	0,8	0,73
Sensibilidade protetora comprometida	0,93	0,93	0,93
Infecção na ferida	0,93	0,93	0,93
Ausente	1,00	1,00	1,00
Presente	1,00	1,00	1,00
Área	1,00	1,00	1,00
Úlcera < 1 cm ^2^	1,00	1,00	0,93
Úlcera ≥ 1 cm ^2^	1,00	1,00	0,93
Profundidade	1,00	1,00	1,00
Úlcera atinge apenas pele e tecido subcutâneo	0,93	0,93	0,93
Úlcera atinge músculo, tendão ou tecido mais profundo	0,86	0,93	0,93
Escore	1,00	0,93	1,00
Escore total possível	1,00	0,93	1,00

*SINBAD = *Site, Ischemia, Neuropathy, Bacterial Infection, Area and Depth*; ^†^CS = Compreensibilidade semântica; ^‡^CC = Compreensibilidade cultural; ^§^UPC = Usabilidade na prática clínica

**Figura 1-:** Versão traduzida e versão preliminar adaptada do SINBAD *System Classification* para o português do Brasil. Goiânia-GO, Belo Horizonte-MG, Jataí-GO, Palmas-TO, São Paulo-SP, Brasil, 2024

**Versão preliminar - traduzida**			**Versão preliminar – adaptada (fase de *cognitive debriefing)***		
Sistema de Avaliação SINBAD*			Sistema SINBAD* para avaliação de úlcera do pé diabético		
Categoria	Definição	Escore	Item de avaliação	Definição	Pontuação
Local	Antepé	0	Local	Antepé	0
	Mediopé e retropé	1		Mediopé e retropé	1
Isquemia	Fluxo sanguíneo sem alteração no pé, com pelo menos um pulso palpável	0	Isquemia	Fluxo sanguíneo arterial sem alteração no pé, com pelo menos um pulso palpável	0
	Evidência clínica de fluxo sanguíneo reduzido no pé	1		Evidência clínica de fluxo sanguíneo arterial reduzido no pé	1
Neuropatia	Sensibilidade protetora intacta	0	Neuropatia	Sensibilidade protetora preservada	0
	Sensibilidade protetora comprometida	1		Sensibilidade protetora comprometida	1
Infecção na ferida	Ausente	0	Infecção na ferida	Ausente	0
	Presente	1		Presente	1
Área	Úlcera < 1 cm ^2^	0	Área	Úlcera < 1 cm ^2^	0
	Úlcera ≥ 1 cm ^2^	1		Úlcera ≥ 1 cm ^2^	1
Profundidade	Úlcera atinge apenas pele e tecido subcutâneo	0	Profundidade	Úlcera atinge apenas pele e tecido subcutâneo	0
	Úlcera atinge músculo, tendão ou tecido mais profundo	1		Úlcera atinge músculo, tendão ou tecido mais profundo	1
**Escore total possível**		**6**	**Pontuação total possível**		**6**

*SINBAD = *Site, Ischemia, Neuropathy, Bacterial Infection, Area and Depth*

Quanto à validação de conteúdo, dos 121 convites enviados, houve retorno de 23 (taxa de resposta de 19,0%). Dentre os respondentes, três foram excluídos por não alcançarem pontuação segundo os critérios de seleção de *experts* adotados neste estudo. Assim, a amostra nesta etapa foi constituída por 20 participantes (10 enfermeiros e 10 médicos), classificados segundo o referencial adotado em *experts* mestres (pontuação entre seis e 20 pontos; n = 3) e *experts* sêniores (pontuação superior a 20 pontos; n = 17), com faixa etária entre 34 e 70 anos, com tempo de trabalho na temática entre 2 e 50 anos.

Na primeira rodada, verificou-se que o sistema SINBAD adaptado para o português brasileiro, alcançou aceitável validade de conteúdo (Tabela 2), mas foram necessários ajustes para alcançar adequada clareza, conforme apontamentos realizados pelos *experts*, especificamente no título e na descrição das opções para os itens local e isquemia. Foram implementadas alterações ao título e ao item isquemia. Todavia, foram mantidas as opções para o item local, de modo a preservar o teor do instrumento original.

Após a implementação das sugestões, a nova versão foi encaminhada para análise dos *experts* em uma segunda rodada, na qual 12 deles enviaram resposta. Com as alterações realizadas, foram alcançados CVR adequados para todos os itens na segunda rodada (Tabela 3).

Mediante a validação conteúdo, foi produzida a versão final do SINBAD *System Classification* para o português do Brasil (Figura 2).

**Tabela 2–:** Validade de conteúdo do Sistema SINBAD* para avaliação de úlcera no pé relacionada ao diabetes - primeira rodada da validação (n = 20). Brasil, 2024

**Itens avaliados**	**n**_e_^†^	**CVR** ^‡^	**CVR** ^‡^ **crítico**
Título: Sistema SINBAD* para avaliação de úlcera do pé diabético	15	0,500	0,500
Local	18	0,800	0,500
Isquemia	16	0,600	0,500
Neuropatia	17	0,700	0,500
Infecção na ferida	18	0,800	0,500
Área	18	0,800	0,500
Profundidade	19	0,900	0,500
Opções para avaliação de Local: (0) antepé (1) mediopé e retropé	15	0,500	0,500
Opções para avaliação de Isquemia: (0) Fluxo sanguíneo arterial sem alteração no pé, com pelo menos um pulso palpável (1) Evidência clínica de fluxo sanguíneo arterial reduzido no pé	13	0,300	0,500
Opções para avaliação de Neuropatia: (0) Sensibilidade protetora preservada (1) Sensibilidade protetora comprometida	16	0,600	0,500
Opções para avaliação de Infecção na ferida: (0) Ausente (1) Presente	17	0,700	0,500
Opções para avaliação de Área: (0) Úlcera < 1 cm ^2^ (1) Úlcera ≥ 1 cm ^2^	17	0,700	0,500
Opções para avaliação de Profundidade: (0) Úlcera atinge apenas a pele e o tecido subcutâneo (1) Úlcera atinge músculo, tendão ou tecido mais profundo	18	0,800	0,500
**Média dos itens**		**0,662**	**0,500**

*SINBAD = *Site, Ischemia, Neuropathy, Bacterial Infection, Area and Depth*; ^†^n_e_ = Número de *experts* que julgaram o item essencial; ^‡^CVR = *Content Validity Ratio*

**Tabela 3-:** Validade de Conteúdo do Sistema SINBAD* para avaliação de úlcera no pé relacionada ao diabetes– segunda rodada da validação (n = 12). Brasil, 2024

**Itens avaliados**	**n**_e_^†^	**CVR** ^‡^	**CVR** ^‡^ **crítico**
Título: Sistema SINBAD* para avaliação de úlcera no pé relacionada ao diabetes	11	0,833	0,667
Local	11	0,833	0,667
Isquemia	11	0,833	0,667
Neuropatia	11	0,833	0,667
Infecção na ferida	11	0,833	0,667
Área	11	0,833	0,667
Profundidade	11	0,833	0,667
Opções para avaliação de Local: (0) antepé (1) mediopé e retropé	12	1,000	0,667
Opções para avaliação de Isquemia: (0) Pé com pelo menos um pulso palpável e sem sinais e/ou sintomas de isquemia (1) Pé com pulsos não palpáveis e/ou presença de sinais ou sintomas de isquemia	11	0,833	0,667
Opções para avaliação de Neuropatia: (0) Sensibilidade protetora preservada (1) Sensibilidade protetora comprometida	11	0,833	0,667
Opções para avaliação de Infecção na ferida: (0) Ausente (1) Presente	11	0,833	0,667
Opções para avaliação de Área: (0) Úlcera < 1 cm ^2^ (1) Úlcera ≥ 1 cm ^2^	11	0,833	0,667
Opções para avaliação de Profundidade: (0) Úlcera atinge apenas a pele e o tecido subcutâneo (1) Úlcera atinge músculo, tendão ou tecido mais profundo	11	0,833	0,667
**Média global dos itens**	**---**	**0,833**	**0,667**

*SINBAD = *Site, Ischemia, Neuropathy, Bacterial Infection, Area and Depth*; ^†^n_e_ = Número de *experts* que julgaram o item essencial; ^‡^CVR = *Content Validity Ratio*

**Figura 2-:** Sistema SINBAD* para avaliação de úlcera no pé relacionada ao diabetes – Versão adaptada do SINBAD *System Classification* para o português do Brasil, 2024

Sistema SINBAD* para avaliação de úlcera no pé relacionada ao diabetes		
Item de avaliação	Definição	Pontuação
Local	Antepé	0
	Mediopé e retropé	1
Isquemia	Pé com pelo menos um pulso palpável e sem sinais e/ou sintomas de isquemia	0
	Pé com pulsos não palpáveis e/ou presença de sinais ou sintomas de isquemia	1
Neuropatia	Sensibilidade protetora preservada	0
	Sensibilidade protetora comprometida	1
Infecção na ferida	Ausente	0
	Presente	1
Área	Úlcera < 1 cm ^2^	0
	Úlcera ≥ 1 cm ^2^	1
Profundidade	Úlcera atinge apenas a pele e o tecido subcutâneo	0
	Úlcera atinge músculo, tendão ou tecido mais profundo	1
**Pontuação total possível**		**6**

*SINBAD = *Site, Ischemia, Neuropathy, Bacterial Infection, Area and Depth*

Para análise de consistência interna participaram 113 pacientes, os quais apresentavam 120 úlceras no pé relacionadas ao diabetes. Entre eles, 29,2 % eram mulheres e 70,8 % eram homens. A idade variou entre 23 e 89 anos, com média igual a 61,23 anos (DP 12,58). O tempo mediano de diagnóstico de diabetes era de 15 anos (IIQ: 10-25 anos) com variação de 1 a 52 anos. A mediana da área das úlceras foi de 2,10 cm² (IIQ: 2,0-24 cm²) com variação de 0,10 cm² e 64,60 cm². A mediana do tempo de lesão foi de 6,50 meses (IIQ: 2-24 meses) com uma variação de 1 a 365 meses. O Sistema SINBAD adaptado para o português do Brasil obteve valor de ômega de McDonald igual a 0,635.

## Discussão

Esta pesquisa representa uma contribuição original visto que, até onde se sabe, pela primeira vez está sendo realizada a adaptação transcultural, validação de conteúdo e avaliação de consistência interna do SINBAD *System Classification*.

A adaptação transcultural seguiu rigorosos padrões metodológicos^(31-33)^, garantindo que a versão traduzida apresentasse equivalência semântica, cultural e usabilidade na prática clínica. Nesse sentido, as modificações durante as etapas de adaptação do instrumento fizeram-se necessárias, atendendo a nuances culturais e regionais para garantir que o instrumento seja compreensível.

O termo *bacterial infection*, adotado originalmente no SINBAD *System,* foi adaptado para *infecção na ferida*, por ser mais apropriado, uma vez que diferentes microrganismos podem ocasionar quadros infecciosos^(39)^. Trata-se de importante fator a ser considerado na avaliação da úlcera, uma vez que sua presença provoca uma resposta local e/ou sistêmica na pessoa, causando dano tecidual local e impedindo a cicatrização^(39)^. Pelas características anatômicas do pé, a perda de integridade da pele e infeções no local assume importância especial, uma vez que podem favorecer processos graves como a osteomielite^(45)^.

A tradução da frase *fluxo sanguíneo intacto no pé*, modificada para *fluxo sanguíneo sem alteração no pé*, justifica-se uma vez que o termo “intacto” se refere a algo que está em perfeitas condições, sem qualquer alteração, o que pode não ser correto afirmar. Clinicamente, o pulso pode estar presente, mas apresentar força e amplitude diminuída, assim, o fluxo está presente, mas não necessariamente intacto. A expressão *sensação de proteção intacta* foi adaptada para *sensibilidade protetora preservada*, uma vez que o termo *sensibilidade protetora* é o que se utiliza no Brasil no contexto da semiologia^(46)^.

A modificação do título da escala para *Sistema de Avaliação SINBAD* é justificável, uma vez que há uma diferença entre instrumentos que apenas pontuam e aqueles que classificam^(47)^. Nesse contexto, é oportuno lembrar que o sistema SINBAD foi originalmente desenvolvido como um sistema de pontuação referente a uma avaliação global da úlcera.

Apesar disso, verificam-se iniciativas de uso da escala com finalidades de classificação, como mostra um estudo em que as úlceras foram classificadas em graves (escore SINBAD 5-6), de grau moderado de risco (escore SINBAD 3-4) e de grau baixo de risco (escore SINBAD 0-2). Nele verificou-se que pacientes com úlceras graves apresentaram maior risco de mortalidade em comparação com aqueles com lesões de grau baixo e moderado^(48)^. Outra iniciativa nesse sentido é a iniciativa da Inglaterra e País de Gales, onde o sistema SINBAD é usado amplamente na auditoria nacional. Naquele contexto, verificou-se que a pontuação SINBAD < 3, resulta na proporção de 60% de cicatrização de úlceras em 12 semanas, e taxa de amputação maior no período de 6 meses de 0,7%. Para pontuação SINBAD ≥ 3 essas taxas são, respectivamente, 35% e 2,7%^(49)^. Contudo, trata-se de iniciativas sem a convergência com a proposta original, que não apresenta classificação^(16)^.

Foram necessárias duas rodadas para alcançar índices aceitáveis para a validade de conteúdo do sistema SINBAD adaptado para o português do Brasil. Apesar de ser uma escala considerada de aplicação fácil e rápida, por ter apenas seis itens de avaliação, seu uso deve ser inequívoco, o que justifica atenção especial a cada detalhe do processo de validação.

O principal destaque foi para o termo *úlcera no pé diabético* que, apesar de possuir uso consolidado na literatura e por entidades internacionais, tais como a Federação Internacional de Diabetes (IDF)^(50)^ e a Organização Mundial de Saúde (OMS)^(51)^, teve recomendação para mudança de terminologia nas diretrizes do IWGDF, publicadas no final de 2023^(1)^, adotando-se *úlcera no pé relacionada ao diabetes,* o que foi amplamente acatado pelos *experts*, que, então, recomendaram a modificação.

A nova terminologia é acompanhada por uma redefinição que amplia a compreensão da doença do pé relacionada ao diabetes^(1)^. Anteriormente, indivíduos com neuropatia periférica ou doença arterial periférica sem úlceras ativas podiam ser erroneamente considerados como não apresentando doença nos pés. A nova definição^(1)^ reconhece que a presença desses fatores de risco já caracteriza uma condição patológica nos pés, mesmo na ausência de ulcerações visíveis. Assim, é fundamental informar aos pacientes que, ao apresentarem neuropatia periférica ou doença arterial periférica nos membros inferiores, eles possuem uma doença em curso nos pés, exigindo intervenções preventivas para evitar complicações mais graves, como o desenvolvimento de úlceras ou infecções.

No Brasil, preconiza-se que a atenção básica resolva 80% dos problemas de saúde da população^(52)^, assim, a prevenção e a avaliação sistemática das úlceras no pé relacionadas ao diabetes representam ações fundamentais, necessitando de profissionais qualificados, capazes de utilizar instrumentos apropriados. Nesse sentido, a versão adaptada do sistema SINBAD de avaliação de úlcera no pé relacionada ao diabetes pode ser uma ferramenta auxiliar de grande importância.

Na consistência interna, obteve-se ômega de McDonald inferior a 0,7, o que sugere que os itens do instrumento não estão medindo o mesmo construto ou fator de forma consistente, o que pode indicar que a escala é multidimensional. Nesse sentido, ressalta-se que ora o SINBAD *SystemClassification* é mencionado como instrumento de avaliação de úlcera no pé relacionada ao diabetes^(29)^, como instrumento de classificação quanto ao potencial de cicatrização^(12)^, e ainda de gravidade da lesão e predição de amputação^(16)^.

Portanto, realizar uma análise fatorial exploratória (AFE) e/ou confirmatória (AFC) pode ajudar a verificar a estrutura fatorial do instrumento e identificar se há múltiplos fatores influenciando o valor da consistência interna^(46)^.

Apesar dessa fragilidade, o sistema tem um grande potencial de uso em todo o território nacional, em especial no contexto da atenção primária, seja para auditoria, a exemplo da Inglaterra a País de Gales, conforme anteriormente citado^(49)^, seja para justificar o referenciamento das pessoas afetadas para serviços especializados, além de servir para monitorar eventos que indicam complicações, tal como o surgimento de infecção, em ambos os casos contribuindo para prevenir amputação^(27)^.

Embora a taxa de resposta tenha sido baixa, considerando o contato inicial para a validação de conteúdo, é importante destacar que foi atendido o perfil e número de *experts* estabelecido na literatura^(35-36)^.

A disponibilização de um sistema adaptado transculturalmente e testado quanto à validade de conteúdo pode contribuir para subsidiar a prática clínica de profissionais que atendem pessoas com úlcera no pé relacionada ao diabetes. Esse sistema possibilita o rastreamento de complicações, o acompanhamento sistematizado e o encaminhamento a serviços especializados, conferindo maior segurança às condutas destinadas a essa população. O instrumento pode favorecer a melhoria do cuidado e do monitoramento das úlceras nos pés relacionadas ao diabetes no Brasil, facilitando a comunicação entre profissionais de saúde e a tomada de decisões clínicas baseadas em evidências.

## Conclusão

O SINBAD *System Classification* está adequadamente adaptado e validado para o português do Brasil, com altos índices de concordância entre os experts em relação à pertinência dos itens. Esta versão está pronta para ser utilizada na prática clínica, constituindo-se em ferramenta válida para a avaliação de úlceras no pé relacionada ao diabetes. No entanto, é importante ressaltar que o sistema ainda apresenta baixa consistência interna, o que torna necessário estudo da validade de construto do instrumento e possível inserção de novos itens.

## Data Availability

Todos os dados gerados ou analisados durante este estudo estão incluídos neste artigo publicado.

# Translation: Adaptación transcultural, validez y confiabilidad del SINBAD *System Classification* al portugués de Brasil*

**DOI:** 10.1590/1518-8345.7768.4721

**Authors:** Julliany Lopes Dias, Eline Lima Borges, Danubia Mendes e Torres, Mônica Antar Gamba, Ângela Lima Pereira, Suelen Gomes Malaquias, Luciana da Silva Barros, Marlene Andrade Martins, Rafael Alves Guimarães, Maria Márcia Bachion

**Affiliations:** 1 Universidade Federal de Goiás, Faculdade de Enfermagem, Goiânia, GO, Brasil. Universidade Federal de Goiás Faculdade de Enfermagem GO Goiânia Brasil; 2 Universidade Federal do Tocantins, Palmas, TO, Brasil. Universidade Federal do Tocantins TO Palmas Brasil; 3 Universidade Federal de Minas Gerais, Escola de Enfermagem, Belo Horizonte, MG, Brasil. Universidade Federal de Minas Gerais Escola de Enfermagem MG Belo Horizonte Brasil; 4 Prefeitura Municipal de Belo Horizonte, Unidade de Referência em Saúde Padre Eustáquio, Belo Horizonte, MG, Brasil. Prefeitura Municipal de Belo Horizonte Unidade de Referência em Saúde Padre Eustáquio MG Belo Horizonte Brasil; 5 Universidade Federal de São Paulo, Escola Paulista de Enfermagem, Departamento de Saúde Coletiva, São Paulo, SP, Brasil. Universidade Federal de São Paulo Escola Paulista de Enfermagem Departamento de Saúde Coletiva SP São Paulo Brasil; 6 Hospital do Coração Anis Rassi, Clínica Médica e Cirúrgica, Goiânia, GO, Brasil. Hospital do Coração Anis Rassi Clínica Médica e Cirúrgica GO Goiânia Brasil; 7 Universidade Federal de Jataí, Jataí, GO, Brasil. Universidade Federal de Jataí GO Jataí Brasil; 8 Becaria del Conselho Nacional de Desenvolvimento Científico e Tecnológico (CNPq), Brasil. Becaria del Conselho Nacional de Desenvolvimento Científico e Tecnológico Brasil

**Keywords:** Traducción, Estudio de Validación, Úlcera del Pie, Pie Diabético, Enfermería, Brasil

## Abstract

realizar la adaptación transcultural de la SINBAD *System Classification* al portugués de Brasil y analizar su validez de contenido y confiabilidad.

estudio clinimétrico que incluyó la adaptación transcultural del sistema SINBAD *System Classification*, empleado para evaluar úlceras en los pies de personas con diabetes. Se verificó la equivalencia semántica, cultural y la usabilidad clínica mediante el índice de concordancia (IC) entre evaluadores (≥ 0,80); la validez de contenido se determinó mediante el *Content Validity Ratio* (CVR) ( p < 0,05), y la consistencia interna mediante el omega de McDonald (> 0,70). La muestra incluyó enfermeros, médicos y personas con úlcera en el pie relacionada con la diabetes. Para la recolección de datos se utilizaron formularios en línea y evaluación clínica presencial de pacientes atendidos en servicios de referencia en Goiânia, Jataí, São Paulo, Belo Horizonte y unidades básicas de salud en Palmas.

participaron 15 enfermeros en el *cognitive debriefing*; 20 expertos (médicos y enfermeros) en la validación de contenido, y 113 pacientes con 120 úlceras en el pie relacionadas con la diabetes. Ocho ítems (términos y expresiones) del SINBAD *System* sufrieron adaptación transcultural. Se obtuvo CVR = 0,84 y omega de McDonald = 0,635.

el sistema SINBAD está adaptado al portugués de Brasil y presenta índices satisfactorios de validez de contenido; sin embargo, requiere inversiones para alcanzar mayor robustez en cuanto a confiabilidad, específicamente en la consistencia interna.

## Destacados

**(1)** El SINBAD *System* se recomienda para la evaluación de la úlcera del pie relacionada con la diabetes. **(2)** La adaptación del SINBAD *System* al portugués de Brasil fue exitosa en este estudio. **(3)** El SINBAD *System* adaptado para Brasil mostró buenas evidencias de validez de contenido. **(4)** Por primera vez se estimó la consistencia interna de la SINBAD *System Classification*. **(5)** El análisis de la consistencia interna del SINBAD *System* indica la necesidad de nuevos estudios.

## Introducción

Las úlceras en los pies relacionadas con la diabetes, anteriormente denominadas úlceras del pie diabético^(1)^, afectan aproximadamente a 26 millones de personas en el mundo cada año^(2)^; representan alrededor del 20% de las hospitalizaciones y son la principal causa de amputaciones no traumáticas^(3)^.

La prevención, mediante intervenciones educativas para el autocuidado, evaluaciones periódicas de los pies con estratificación del riesgo de ulceración e implementación de estrategias basadas en evidencias para la gestión de factores de riesgo, es indispensable para reducir la incidencia de estas úlceras y prevenir amputaciones^(4)^.

Una vez instaurada la úlcera, es necesario establecer acciones de tratamiento efectivas, acompañar la evolución y detectar precozmente complicaciones. En este contexto, la utilización de sistemas de clasificación de úlceras en el pie relacionadas con la diabetes es de gran importancia^(4-5)^. Estos sistemas deben integrarse en la evaluación clínica global del paciente como estrategia de seguimiento de la cicatrización, riesgo de amputación y manejo clínico, además de ser útiles en protocolos de estudios epidemiológicos y clínicos en el área.

Existe una variedad de sistemas para evaluar la cicatrización de úlceras en los pies relacionadas con la diabetes, incluidos el sistema Meggitt-Wagner^(6)^; Puntuación de la Universidad del Texas (UT)^(7)^; Sistema S(AD)SAD - *Size (Area and Depth), infection (Sepsis), ischemia (Arteriopathy), and denervation (Neuropathy)*^(8)^; Foster y Edmonds^(9)^; Van Acker/Peters^(10)^; Margolis^(11)^; Sistema PEDIS - *Perfusion, Extent, Depth, Infection, Sensation*^(12)^; Sistema DEPA - *depth of the ulcer, extent of bacterial colonization, phase of ulcer healing and the associated underlying etiology*^(13)^; Sistema CHS - *Curative Health Services*^(14)^; Sistema DUSS - *Diabetic Ulcer Severity Score*^(15)^; Sistema de Clasificación SINBAD - *Site, Ischemia, Neuropathy, Bacterial Infection, Area and Depth*^(16)^; MAID - *Multiple, Ankle Pulse absent, Infection, Duration*^(17)^; SEWSS - *Saint Elian Wound Score System*^(18)^; WifI - *Wound, Ischemia, and Infection*^(19)^ y DIAFORA - *Diabetic Foot Risk Assessment*^(20)^.

Sin embargo, dependiendo del contexto o propósito de uso, todos estos sistemas presentan limitaciones. No existe un patrón oro, por lo que la elección se realiza de acuerdo con el objetivo del profesional y/o de la investigación científica. La mayoría de los sistemas se centran solo en la patología local de las úlceras en el pie relacionadas con la diabetes y no evalúan adecuadamente todos los parámetros relevantes para la cicatrización de la úlcera^(5)^.

En Brasil, el Ministerio de Salud adopta la Puntuación *University of Texas* (UT) y la pone a disposición en una versión traducida, sin detallar el proceso de traducción y adaptación transcultural^(21)^. Los sistemas Wagner, S(AD)SAD y UT se utilizaron en un estudio comparativo realizado en Brasil^(22)^, en el que tampoco se menciona adaptación ni validación.

El consenso de la *Sociedade Brasileira de Angiologia e de Cirurgia Vascular*^(23)^ y la versión traducida de la guía del *International Working Group on the Diabetic Foot* (IWGDF)^(24)^ presentan traducciones simplificadas de clasificaciones internacionales, como WIfI y SINBAD, respectivamente. No obstante, no llevaron a cabo un proceso formal de traducción y adaptación transcultural, lo que puede comprometer la precisión y la aplicabilidad de los sistemas en el contexto brasileño.

Aunque WIfI y SINBAD son recomendados por el IWGDF^(24)^ para la evaluación de úlceras en los pies relacionadas con la diabetes, SINBAD se destaca por ser un instrumento conciso y por incluir la evaluación de la neuropatía, aspecto no contemplado por WIfI. Esta característica hace que SINBAD sea particularmente útil para la comunicación entre profesionales de la salud, el cribado de casos más graves y la derivación a equipos especializados^(5)^.

En Brasil, la atención a las personas con úlcera en el pie relacionada con la diabetes está asegurada por el Sistema Único de Salud (SUS) en todos los niveles de atención, entre los cuales destaca la Atención Primaria de Salud en la prevención, la identificación de complicaciones y las derivaciones necesarias a servicios de referencia^(21)^. En este proceso, es esencial el uso de instrumentos de evaluación para el cribado, el seguimiento de la evolución, la toma de decisiones clínicas y la derivación adecuada.

El sistema SINBAD es una simplificación de S(AD)SAD^(8)^ y posee seis elementos de evaluación: *Site, Ischemia, Neuropathy, Bacterial Infection, Area and Depth*, que forman el acrónimo SINBAD^(16)^. La evaluación de estos ítems resulta en una puntuación binaria (0 o 1) y un puntaje máximo de seis puntos, lo que hace que este sistema sea de fácil comprensión y aplicación en la práctica clínica^(5)^.

El SINBAD *System Classification*^(16)^ se considera uno de los sistemas más simples y completos^(24)^, con estudios efectuados en el exterior que demuestran una buena reproducibilidad interobservador^(25-26)^. Además, SINBAD se ha asociado con factores predictivos de amputación, lo que refuerza su utilidad clínica^(16,27-28)^.

Sin embargo, hasta el momento no existen registros de su adaptación transcultural a otros idiomas, incluido el portugués de Brasil. Además, no se identificaron estudios que investiguen su consistencia interna^(29)^ o validez de contenido, aspectos fundamentales para garantizar la aplicabilidad y precisión del instrumento en diferentes contextos culturales y lingüísticos. La ausencia de estas evidencias limita su utilización en poblaciones que no hablan el idioma original del instrumento, lo que destaca la necesidad de estudios que aborden estos aspectos para ampliar su aplicabilidad global.

La realización de este estudio se justifica por la necesidad de disponer de un sistema con evidencias de validez y confiabilidad para evaluar la úlcera en el pie relacionada con la diabetes, adecuado a la realidad brasileña, que incluya factores ligados al paciente, a la úlcera y a las complicaciones, sea de fácil comprensión y aplicación y pueda utilizarse en la práctica clínica, permitiendo decisiones precisas por parte de los profesionales que atienden a personas con diabetes, especialmente en la atención primaria de salud. Así, el objetivo de este estudio es realizar la adaptación transcultural de la SINBAD *System Classification* al portugués de Brasil y analizar su validez de contenido y confiabilidad.

## Método

### Diseño

Estudio clinimétrico^(30)^ multicéntrico, desarrollado con foco en la adaptación transcultural, la validación de contenido y la confiabilidad en relación con el requisito de consistencia interna del SINBAD *System Classification*^(16)^.

### Contexto

El estudio se llevó a cabo entre octubre de 2022 y julio de 2024. Los lugares del estudio incluyeron el entorno virtual, para las etapas de adaptación transcultural y validación de contenido.

Para la consistencia interna, los escenarios fueron cinco servicios ambulatorios de referencia para la atención a personas con úlcera en el pie relacionada con la diabetes, dos en Goiânia-GO, uno en Jataí-GO, uno en São Paulo-SP y uno en Belo Horizonte-MG, además de diez unidades básicas de salud en Palmas-TO.

Debido a la multiplicidad de etapas y procedimientos involucrados, para una mejor comprensión del diseño se presentarán de forma agrupada informaciones como población, muestra, criterios de inclusión y exclusión, fuentes, recolección y organización de datos adoptados en cada una de ellas.

### Adaptación transcultural

La adaptación transcultural de la SINBAD *System Classification* se basó en *guidelines* internacionales y siguió seis etapas: traducción inicial, síntesis de las traducciones, retrotraducción, revisión por el comité de especialistas y *cognitive debriefing*^(31-33)^. Se llevó a cabo de octubre de 2022 a junio de 2023, con la autorización previa del autor correspondiente de la publicación original.

Inicialmente, los 23 componentes del sistema (términos/frases) fueron traducidos del inglés al portugués de Brasil por una empresa especializada que siguió criterios de selección de traductores conforme a los *guidelines* de referencia^(31-33)^. Se indicaron así dos traductores independientes bilingües (inglés-portugués), cuya lengua materna era el portugués, uno de ellos conocedor de la terminología técnica del área y el otro sin dominio de la temática. En la síntesis de las traducciones, los traductores y el comité de especialistas llegaron a un consenso sobre las divergencias y discutieron la adopción de términos adecuados al contexto del área y de Brasil. El comité de especialistas estuvo compuesto por cinco doctores, enfermeros con *expertise* en úlceras en los pies relacionadas con la diabetes, un profesional de idiomas y los dos traductores implicados. La interacción con el autor correspondiente del estudio original se realizó mediante correos electrónicos y reuniones virtuales. El comité de especialistas revisó las traducciones y desarrolló la versión del instrumento, que pasó por el proceso de retrotraducción, efectuada por dos traductores nativos en inglés, cegados respecto a la versión original, obteniéndose la versión que se sometería a la siguiente etapa de la adaptación.

Para evaluar la comprensión y la equivalencia de la traducción a términos de la cultura brasileña e identificar posibles problemas en relación con el uso en la práctica clínica, se llevó a cabo la etapa de *cognitive debriefing*^(33)^ con enfermeros, seleccionados mediante la técnica de bola de nieve^(34)^ a partir de la invitación inicial de los investigadores. Para la selección de los profesionales e inclusión en la muestra como jueces en esta etapa, el requisito esencial era presentar una experiencia mínima de seis meses en la atención a personas con úlcera en el pie relacionada con la diabetes.

Para la recolección de datos de esta etapa se utilizó un formulario en línea estructurado en *Google Forms*^®^, disponible por correo electrónico. El instrumento incluyó ítems de caracterización profesiográfica de los participantes (área de graduación, titulación y tiempo de experiencia) y la versión traducida de la SINBAD *System Classification.* Los jueces evaluaron la comprensibilidad semántica, cultural y la usabilidad clínica de los ítems mediante las opciones ausente, parcial o total. Los comentarios y sugerencias enviados por los participantes y los ítems que presentaron un índice de concordancia inferior al valor de referencia (≥ 80,0%) fueron revisados por el comité de especialistas. Las modificaciones consideradas pertinentes por el comité se incorporaron a la escala para su posterior sometimiento al proceso de verificación de validez de contenido.

### Validez de contenido

Para la evaluación de la validez de contenido se conformó un comité de expertos^(35)^, reclutados mediante muestreo no probabilístico intencional, de julio a septiembre de 2023, utilizando la Plataforma Lattes (*Conselho Nacional de Desenvolvimento Científico e Tecnológico* – Brasil) y la técnica de bola de nieve^(34)^.

El criterio de inclusión para participar como experto en la evaluación de la validez de contenido fue alcanzar una puntuación mínima de cinco puntos en el sistema de puntuación^(36)^, que considera experiencia clínica, actuación en la enseñanza, investigación y publicaciones en el área, además de la titulación académica, según se detalla a continuación:

*Experiencia clínica*: otorga 4 puntos cuando el profesional posee al menos dos años de atención a personas con diabetes y úlceras en el pie

*Enseñanza*: participación en actividades docentes, con enfoque en el manejo de úlceras en el pie relacionadas con la diabetes durante al menos un año, otorga 1 punto.

*Formación académica*: concede 2 puntos a quienes poseen doctorado en el área de diabetes y úlceras en el pie, 1 punto para maestría y 1 punto para residencias o especializaciones afines, como cirugía vascular, ortopedia, endocrinología, dermatología o tratamiento de heridas

En esta etapa se utilizó, para la recolección de datos, el mismo instrumento de caracterización profesiográfica de la fase anterior y, para la evaluación de la pertinencia o representatividad de los ítems, una escala tipo Likert con las opciones: 1) completamente insatisfactoria, 2) muy poco satisfactoria, 3) tal vez de algún modo satisfactoria, 4) satisfactoria, 5) muy satisfactoria. Este instrumento fue adaptado a partir de la propuesta de Coluci, Alexandre y Milani^(37)^, estructurado en *Google Forms*^®^ y enviado por correo electrónico a 40 profesionales considerados elegibles según la información de sus currículos y a 81 indicados mediante la técnica de bola de nieve^(34)^.

La confirmación del cumplimiento de los criterios de inclusión se realizó a partir de las respuestas indicadas en el formulario de caracterización profesiográfica.

### Confiabilidad

El tamaño muestral para verificar la consistencia interna se estimó en al menos 100 pacientes, conforme a las recomendaciones para escalas compuestas por menos de 10 ítems^(38)^. El muestreo no probabilístico por conveniencia involucró a individuos con úlcera en el pie relacionada con la diabetes, atendidos en los escenarios de investigación, con edad igual o superior a 18 años, independientemente del tiempo de diagnóstico de diabetes o de evolución de la úlcera.

Para la recolección de datos se efectuó una entrevista breve, guiada por un guion estructurado que incluía datos sociodemográficos y clínicos de los pacientes; posteriormente se aplicó la SINBAD *System Classification* para la evaluación de la úlcera en el pie.

Si había más de una úlcera en el mismo pie, se evaluó la considerada más grave según profundidad, signos de infección y área; en caso de úlcera en ambos pies, cada una se evaluó como un evento distinto, de modo que un paciente podía tener dos lesiones evaluadas, una en cada pie, aplicando para cada una los formularios correspondientes al SINBAD *System Classification.*

Todos los investigadores de campo participaron en una capacitación presencial de 30 minutos con actividades prácticas y lectura y discusión de una guía de uso del instrumento.

Los pacientes fueron evaluados después de la limpieza y desbridamiento de la úlcera (cuando fue necesario) durante la atención de rutina. Para la evaluación de los ítems y la puntuación del sistema se adoptaron las recomendaciones del instrumento original^(16)^.

El ítem *sitio* se evaluó mediante inspección e identificación de la región anatómica del pie donde se presentaba la úlcera^(16)^. En el ítem *isquemia* se valoró el flujo sanguíneo arterial de la pierna afectada mediante palpación de los pulsos de la arteria pedía y tibial posterior y análisis de la historia clínica del paciente^(16)^, además de pruebas adicionales como la maniobra de *isquemia* provocada y el tiempo de relleno capilar.

Para evaluar el ítem *neuropatía* se realizó la prueba con monofilamento de nylon 5.07 o 10 g, conforme a la literatura^(16,24)^. En cuanto al ítem *infección en la herida*, se consideraron los signos clínicos de infección en tejido blando u óseo según las directrices de la *Infectious Diseases Society of America* y del *International Working Group on the Diabetic Foot* (IWGDF/IDSA)^(4,16)^ y del *International Wound Infection Institute* (IWII)^(39)^.

Para el ítem área se midieron las dos dimensiones máximas del diámetro en ángulos rectos con una regla de papel desechable y estéril, multiplicándose (longitud × anchura) para calcular el área de la úlcera^(16)^. La *profundidad* se evaluó mediante la observación de los tejidos afectados e inspección del lecho de la herida para visualizar las estructuras comprometidas, como epidermis, dermis, tejido subcutáneo, músculos, tendones u otros tejidos más profundos^(16)^.

### Tratamiento y análisis de los datos

En el análisis descriptivo de los datos profesiográficos, sociodemográficos y clínicos de los participantes, para las variables cualitativas se utilizaron frecuencias absolutas y relativas. Para las variables cuantitativas se calcularon medidas de tendencia central (media o mediana) y dispersión (desviación estándar – DE o rango intercuartílico – RIC), según la normalidad o no de la distribución. La normalidad de las variables cuantitativas se verificó mediante la prueba Kolmogorov-Smirnov con corrección de Lilliefors (n>30).

El Índice de Concordancia (IC) entre los jueces en la etapa de *cognitive debriefing* se calculó considerando el número de acuerdos (A), es decir, coincidencias para la opción *total* en la evaluación de la comprensibilidad semántica, cultural y la usabilidad clínica de los ítems, dividido por el número de acuerdos (A) más los desacuerdos (D), que indicaban el número de jueces que participaron en la etapa, mediante la siguiente fórmula:

$$IC=\frac{A}{A+D}\;\left(1\right)$$

Se consideraron aceptables los índices iguales o superiores a 0,80^(40)^.

Para estimar la validez de contenido se calculó el CVR (*Content Validity Ratio*), cuyo valor esperado depende del número de expertos^(41)^, utilizando la fórmula:

$$CVR=\frac{n-\frac{N}{2}}{\frac{N}{2}}\;\left(2\right)$$

donde *n* es el número de expertos que consideran que el ítem es *satisfactorio (4)* o *muy satisfactorio (5)*, y *N* es el número de expertos que evaluaron el contenido.

El valor obtenido se comparó con el valor crítico de CVR para una significancia de 0,05^(42)^ y se consideró válido el valor igual o superior al CVR crítico.

Para el análisis de la consistencia interna se empleó el omega de McDonald, aceptándose valores > 0,70^(43)^.

Todos los datos de la investigación se introdujeron en la plataforma *Research Electronic Data Capture* (REDCap) de la *Vanderbilt University*, se exportaron al *software Microsoft Excel*^®^ versión 2408 y, de este, al IBM SPSS *Statistics* versión 28.0 para los análisis descriptivos, y al programa R 4.1.2^(44)^ para calcular el omega de McDonald.

### Aspectos éticos

Este estudio fue aprobado por el Comité de Ética en Investigación (CAAE: 69265323.0.1001.5078).

## Resultados

En la adaptación transcultural del SINBAD *System Classification*, ocho de los 23 componentes (términos/frases) analizados sufrieron modificaciones para atender a la comprensión idiomática, cultural y la usabilidad en la práctica clínica.

Las primeras modificaciones se produjeron en la fase de síntesis de las traducciones, por parte del comité de especialistas, en la que el ítem *bacterial infection*, traducido como *infección bacteriana*, se adaptó a infección en la herida. Además, el comité de especialistas propuso la adaptación del título de la escala, traducido como *Sistema de Clasificación SINBAD* a *Sistema de Evaluación SINBAD*, dado que el sistema no permite la clasificación, sino una puntuación referente a la evaluación global de la úlcera.

Se registraron divergencias de traducción relativas al uso de términos diferentes, aunque sinónimos, que se resolvieron en reunión del comité de especialistas. Se eligieron los términos que mejor respondían al contexto clínico brasileño. Como ejemplo se citan los términos *score* y puntuación, entre los cuales se optó por el segundo. Otros ítems se tradujeron de forma idéntica, pero precisaron adaptación, tales como *flujo sanguíneo intacto en el pie*, que se adaptó a *flujo sanguíneo sin alteración en el pie*, y *sensación de protección*, adaptada a *sensibilidad protectora*.

En la retrotraducción se obtuvo una versión con el mismo sentido que la versión original del sistema SINBAD. El material traducido, sintetizado y retrotraducido se discutió con el autor correspondiente, William Jeffcoate, quien estuvo de acuerdo con las modificaciones, llegándose así a la versión prefinal, que se sometería al *cognitive debriefing*.

Participaron como jueces en el *cognitive debriefing* 15 enfermeros, con edad entre 25 y 65 años y tiempo de actuación en el área entre 3 y 40 años. La mayoría tenía titulación de especialización en el área de dermatología o estomaterapia (n = 7) y maestría (n = 5).

La comprensibilidad semántica, cultural y la usabilidad en la práctica clínica de la versión traducida del sistema SINBAD alcanzó índices satisfactorios de concordancia entre los jueces para todos los ítems (Tabla 1), excepto para la descripción de una de las opciones del resultado de la evaluación de isquemia: *Evidencia clínica de flujo sanguíneo reducido en el pie*, y para la descripción de una de las opciones del resultado de la evaluación de neuropatía: *Sensibilidad protectora intacta*.

En el *cognitive debriefing* los jueces señalaron modificaciones, las cuales fueron discutidas por el comité de especialistas y luego implementadas, generándose una versión adaptada preliminarmente (Figura 1).

Con respecto a la validación de contenido, de las 121 invitaciones enviadas se recibieron 23 respuestas (tasa de respuesta del 19,0%). Entre los respondientes, tres fueron excluidos por no alcanzar la puntuación exigida por los criterios de selección de expertos adoptados en este estudio. Así, la muestra en esta etapa quedó conformada por 20 participantes (10 enfermeros y 10 médicos), clasificados según el referente adoptado en expertos maestros (puntuación entre seis y 20 puntos; n = 3) y expertos séniores (puntuación superior a 20 puntos; n = 17), con edades entre 34 y 70 años y tiempo de trabajo en la temática entre 2 y 50 años.

**Tabla 1-:** Índice de concordancia entre los jueces sobre la comprensibilidad semántica, cultural y la usabilidad en la práctica clínica de los términos/frases del sistema SINBAD (n = 15). Goiânia-GO, Belo Horizonte-MG, Jataí-GO, Palmas-TO, São Paulo-SP, Brasil, 2024

Términos/frases del SINBAD* *System Classification*	**CS** ^†^	**CC** ^‡^	**UPC** ^§^
Sistema de Evaluación SINBAD*	0,86	0,86	0,93
Categoría	1,00	0,93	0,93
Definición	0,93	0,93	0,93
Sitio	0,8	0,86	0,86
Antepié	0,93	0,86	0,93
Mediopié y retropié	0,93	0,86	0,93
Isquemia	1,00	1,00	1,00
Flujo sanguíneo sin alteración en el pie, con al menos un pulso palpable	0,93	0,86	0,86
Evidencia clínica de flujo sanguíneo reducido en el pie	0,80	0,86	0,73
Neuropatía	1,00	1,00	1,00
Sensibilidad protectora intacta	0,73	0,8	0,73
Sensibilidad protectora comprometida	0,93	0,93	0,93
Infección en la herida	0,93	0,93	0,93
Ausente	1,00	1,00	1,00
Presente	1,00	1,00	1,00
Área	1,00	1,00	1,00
Úlcera < 1 cm ^2^	1,00	1,00	0,93
Úlcera ≥ 1 cm ^2^	1,00	1,00	0,93
Profundidad	1,00	1,00	1,00
Úlcera que alcanza solo piel y tejido subcutáneo	0,93	0,93	0,93
Úlcera que alcanza músculo, tendón o tejido más profundo	0,86	0,93	0,93
Escore	1,00	0,93	1,00
Escore total posible	1,00	0,93	1,00

*SINBAD = *Site, Ischemia, Neuropathy, Bacterial Infection, Area and Depth*; ^†^CS = Comprensibilidad semántica; ^‡^CC = Compresibilidad cultural; ^§^UPC = Usabilidad en la práctica clínica

**Figura 1-:** Versión traducida y versión preliminar adaptada de la SINBAD *System Classification* al portugués de Brasil. Goiânia-GO, Belo Horizonte-MG, Jataí-GO, Palmas-TO, São Paulo-SP, Brasil, 2024

**Versión preliminar – traducida**			**Versión preliminar – adaptada (fase de *cognitive debriefing)***		
Sistema de Evaluación SINBAD*			Sistema SINBAD* para la evaluación de la úlcera del pie diabético		
Categoría	Definición	Escore	Ítem de evaluación	Definición	Puntuación
Sitio	Antepié	0	Sitio	Antepié	0
	Mediopié y retropié	1		Mediopié y retropié	1
Isquemia	Flujo sanguíneo sin alteración en el pie, con al menos un pulso palpable	0	Isquemia	Flujo sanguíneo arterial sin alteración en el pie, con al menos un pulso palpable	0
	Evidencia clínica de flujo sanguíneo reducido en el pie	1		Evidencia clínica de flujo sanguíneo arterial reducido en el pie	1
Neuropatía	Sensibilidad protectora preservada	0	Neuropatía	Sensibilidad protectora preservada	0
	Sensibilidad protectora comprometida	1		Sensibilidad protectora comprometida	1
Infección en la herida	Ausente	0	Infección en la herida	Ausente	0
	Presente	1		Presente	1
Área	Úlcera < 1 cm ^2^	0	Área	Úlcera < 1 cm ^2^	0
	Úlcera ≥ 1 cm ^2^	1		Úlcera ≥ 1 cm ^2^	1
Profundidad	Úlcera que alcanza solo piel y tejido subcutáneo	0	Profundidad	Úlcera que alcanza solo piel y tejido subcutáneo	0
	Úlcera que alcanza músculo, tendón o tejido más profundo	1		Úlcera que alcanza músculo, tendón o tejido más profundo	1
**Escore total posible**		**6**	**Puntuación total posible**		**6**

*SINBAD = *Site, Ischemia, Neuropathy, Bacterial Infection, Area and Depth*

En la primera ronda se comprobó que el sistema SINBAD adaptado al portugués brasileño alcanzó validez de contenido aceptable (Tabla 2), pero fueron necesarios ajustes para lograr claridad adecuada, según las observaciones de los expertos, específicamente en el título y en la descripción de las opciones para los ítems local e isquemia. Se implementaron modificaciones en el título y en el ítem isquemia. Sin embargo, se mantuvieron las opciones para el ítem local, a fin de preservar el contenido del instrumento original.

Después de la implementación de las sugerencias, la nueva versión fue enviada para el análisis de los expertos en una segunda ronda, en la que 12 de ellos enviaron respuesta. Con las modificaciones realizadas, se alcanzaron valores de CVR adecuados para todos los ítems en la segunda ronda (Tabla 3).

Tras la validación de contenido, se elaboró la versión final de la SINBAD *System Classification* en portugués de Brasil (Figura 2).

**Tabla 2-:** Validez de contenido del Sistema SINBAD* para la evaluación de la úlcera en el pie relacionada con la diabetes: primera ronda de la validación (n = 20). Brasil, 2024

Ítems evaluados	**n**_e_^†^	**CVR** ^‡^	**CVR** ^‡^ **crítico**
Título: Sistema SINBAD* para la evaluación de úlcera del pie diabético	15	0,500	0,500
Sito	18	0,800	0,500
Isquemia	16	0,600	0,500
Neuropatía	17	0,700	0,500
Infección en la herida	18	0,800	0,500
Área	18	0,800	0,500
Profundidad	19	0,900	0,500
Opciones para la evaluación de sitio: (0) antepié (1) mediopié y retropié	15	0,500	0,500
Opciones para la evaluación de Isquemia: (0) Flujo sanguíneo arterial sin alteración en el pie, con al menos un pulso palpable (1) Evidencia clínica de flujo sanguíneo arterial reducido en el pie	13	0,300	0,500
Opciones para la evaluación de Neuropatía: (0) Sensibilidad protectora preservada (1) Sensibilidad protectora comprometida	16	0,600	0,500
Opciones para la evaluación de Infección en la herida: (0) Ausente (1) Presente	17	0,700	0,500
Opciones para la evaluación de Área: (0) Úlcera < 1 cm2 (1) Úlcera ≥ 1 cm2	17	0,700	0,500
Opciones para la evaluación de Profundidad: (0) Úlcera que alcanza solo la piel y el tejido subcutáneo (1) Úlcera que alcanza músculo, tendón o tejido más profundo	18	0,800	0,500
**Media de los ítems**		**0,662**	**0,500**

*SINBAD = *Site, Ischemia, Neuropathy, Bacterial Infection, Area and Depth*; ^†^n_e_ = Número de expertos que juzgaron el ítem esencial; ^‡^CVR = *Content Validity Ratio*

**Tabla 3-:** Validez de contenido del Sistema SINBAD* para la evaluación de la úlcera en el pie relacionada con la diabetes – segunda ronda de la validación (n = 12). Brasil, 2024

Ítems evaluados	**n**_e_^†^	**CVR** ^‡^	**CVR** ^‡^ **crítico**
Título: Sistema SINBAD* para la evaluación de úlcera en el pie relacionada con la diabetes	11	0,833	0,667
Sitio	11	0,833	0,667
Isquemia	11	0,833	0,667
Neuropatía	11	0,833	0,667
Infección en la herida	11	0,833	0,667
Área	11	0,833	0,667
Profundidad	11	0,833	0,667
Opciones para la evaluación de sitio: (0) antepié (1) mediopié y retropié	12	1,000	0,667
Opciones para la evaluación de Isquemia: (0) Pie con al menos un pulso palpable y sin signos y/o síntomas de isquemia (1) Pie con pulsos no palpables y/o presencia de signos o síntomas de isquemia	11	0,833	0,667
Opciones para la evaluación de Neuropatía: (0) Sensibilidad protectora preservada (1) Sensibilidad protectora comprometida	11	0,833	0,667
Opciones para la evaluación de Infección en la herida: (0) Ausente (1) Presente	11	0,833	0,667
Opciones para la evaluación de Área: (0) Úlcera < 1 cm ^2^ (1) Úlcera ≥ 1 cm ^2^	11	0,833	0,667
Opciones para la evaluación de profundidad: (0) Úlcera que alcanza solo la piel y el tejido subcutáneo (1) Úlcera que alcanza músculo, tendón o tejido más profundo	11	0,833	0,667
**Media global de los ítems**	**---**	**0,833**	**0,667**

*SINBAD = *Site, Ischemia, Neuropathy, Bacterial Infection, Area and Depth*; ^†^n_e_ = Número de expertos que juzgaron el ítem esencial; ^‡^CVR = *Content Validity Ratio*

**Figura 2-:** Sistema SINBAD* para la evaluación de la úlcera en el pie relacionada con la diabetes – Versión adaptada de la SINBAD *System Classification* al portugués de Brasil, 2024

Sistema SINBAD* para la evaluación de la úlcera en el pie relacionada con la diabetes		
Ítem de evaluación	Definición	Puntuación
Sitio	Antepié	0
	Mediopié y retropié	1
Isquemia	Pie con al menos un pulso palpable y sin signos y/o síntomas de isquemia	0
	Pie con pulsos no palpables y/o presencia de signos o síntomas de isquemia	1
Neuropatía	Sensibilidad protectora preservada	0
	Sensibilidad protectora comprometida	1
Infección en la herida	Ausente	0
	Presente	1
Área	Úlcera < 1 cm ^2^	0
	Úlcera ≥ 1 cm ^2^	1
Profundidad	Úlcera que alcanza solo la piel y el tejido subcutáneo	0
	Úlcera que alcanza músculo, tendón o tejido más profundo	1
**Puntuación total posible**		**6**

*SINBAD = *Site, Ischemia, Neuropathy, Bacterial Infection, Area and Depth*

Para el análisis de consistencia interna participaron 113 pacientes, quienes presentaban 120 úlceras en el pie relacionadas con la diabetes. De ellos, el 29,2% eran mujeres y el 70,8% hombres. La edad osciló entre 23 y 89 años, con una media de 61,23 años (DE 12,58). La mediana del tiempo de diagnóstico de diabetes fue de 15 años (RIC: 10-25 años), con variación de 1 a 52 años. La mediana del área de las úlceras fue de 2,10 cm² (RIC: 2,0-24 cm²), con variación de 0,10 cm² a 64,60 cm². La mediana del tiempo de la lesión fue de 6,50 meses (RIC: 2-24 meses), con una variación de 1 a 365 meses. El Sistema SINBAD adaptado al portugués de Brasil obtuvo un valor de omega de McDonald igual a 0,635.

## Discusión

Esta investigación representa una contribución original, pues, hasta donde se sabe, es la primera vez que se realiza la adaptación transcultural, la validación de contenido y la evaluación de la consistencia interna de la SINBAD *System Classification*.

La adaptación transcultural siguió rigurosos estándares metodológicos^(31-33)^, garantizando que la versión traducida presentara equivalencia semántica, cultural y usabilidad en la práctica clínica. En este sentido, las modificaciones durante las etapas de adaptación del instrumento fueron necesarias para atender matices culturales y regionales y asegurar que el instrumento resulte comprensible.

La modificación del término *bacterial infection* adoptado originalmente en el SINBAD *System* se adaptó a infección en la herida, por resultar más apropiado, ya que diferentes microorganismos pueden ocasionar cuadros infecciosos^(39)^. Se trata de un factor importante a considerar en la evaluación de la úlcera, dado que su presencia provoca una respuesta local y/o sistémica en la persona, causa daño tisular local e impide la cicatrización^(39)^. Por las características anatómicas del pie, la pérdida de integridad de la piel y las infecciones en el sitio adquieren especial relevancia, pues pueden favorecer procesos graves como la osteomielitis^(45)^.

La traducción de la frase *flujo sanguíneo intacto en el pie*, modificada a *flujo sanguíneo sin alteración en el pie*, se justifica porque el término intacto se refiere a algo en perfectas condiciones, sin alteraciones, lo que puede no ser correcto afirmar. Clínicamente, el pulso puede estar presente, pero con fuerza y amplitud disminuidas; así, el flujo está presente, aunque no necesariamente intacto. La expresión *sensación de protección intacta* se adaptó a *sensibilidad protectora preservada*, puesto que el término *sensibilidad protectora* es el utilizado en Brasil en el contexto de la semiología^(46)^.

La modificación del título de la escala a *Sistema de Avaliação SINBAD* se justifica, ya que existe una diferencia entre los instrumentos que solo otorgan puntuación y aquellos que clasifican^(47)^. En este contexto, es oportuno recordar que el sistema SINBAD fue desarrollado originalmente como un sistema de puntuación referente a una evaluación global de la úlcera.

Aun así, se observan iniciativas de uso de la escala con fines de clasificación, como muestra un estudio en el que las úlceras fueron clasificadas como graves (puntuación SINBAD 5-6), de riesgo moderado (puntuación SINBAD 3-4) y de bajo riesgo (puntuación SINBAD 0-2). En dicho estudio se comprobó que los pacientes con úlceras graves presentaron mayor riesgo de mortalidad en comparación con aquellos con lesiones de grado bajo y moderado^(48)^. Otra iniciativa en este sentido es la emprendida en Inglaterra y Gales, donde el sistema SINBAD se utiliza ampliamente en la auditoría nacional. En ese contexto se verificó que una puntuación SINBAD < 3 resulta en una proporción del 60% de cicatrización de úlceras en 12 semanas y una tasa de amputación mayor en un periodo de 6 meses del 0,7%. Para puntuación SINBAD ≥ 3 estas tasas son, respectivamente, 35% y 2,7%^(49)^. Sin embargo, se trata de iniciativas sin convergencia con la propuesta original, que no presenta clasificación^(16)^.

Fueron necesarias dos rondas para alcanzar índices aceptables de validez de contenido del sistema SINBAD adaptado al portugués de Brasil. Aunque se trata de una escala considerada de aplicación fácil y rápida, al tener solo seis ítems de evaluación, su uso debe ser inequívoco, lo que justifica una atención especial a cada detalle del proceso de validación.

El principal destaque fue el término *úlcera en el pie diabético* que, a pesar de estar consolidado en la literatura y por entidades internacionales, como la Federación Internacional de Diabetes (IDF)^(50)^ y la Organización Mundial de la Salud (OMS)^(51)^, recibió la recomendación de cambio de terminología en las directrices del IWGDF, publicadas a finales de 2023^(1)^, adoptándose *úlcera en el pie relacionada con la diabetes*, lo que fue ampliamente aceptado por los expertos, quienes recomendaron la modificación.

La nueva terminología va acompañada de una redefinición que amplía la comprensión de la enfermedad del pie relacionada con la diabetes^(1)^. Anteriormente, los individuos con neuropatía periférica o enfermedad arterial periférica sin úlceras activas podían considerarse erróneamente como no portadores de enfermedad en los pies. La nueva definición^(1)^ reconoce que la presencia de estos factores de riesgo ya caracteriza una condición patológica en los pies, incluso sin ulceraciones visibles. Así, es fundamental informar a los pacientes que, al presentar neuropatía periférica o enfermedad arterial periférica en los miembros inferiores, poseen una enfermedad en curso en los pies que exige intervenciones preventivas para evitar complicaciones más graves, como el desarrollo de úlceras o infecciones.

En Brasil se recomienda que la atención básica resuelva el 80% de los problemas de salud de la población^(52)^; por ello, la prevención y la evaluación sistemática de las úlceras en el pie relacionadas con la diabetes representan acciones fundamentales que requieren profesionales cualificados capaces de utilizar instrumentos apropiados. En este sentido, la versión adaptada del sistema SINBAD para la evaluación de la úlcera en el pie relacionada con la diabetes puede ser una herramienta auxiliar de gran importancia.

En la consistencia interna se obtuvo un omega de McDonald inferior a 0,7, lo que sugiere que los ítems del instrumento no miden el mismo constructo o factor de forma consistente, lo que puede indicar que la escala es multidimensional. En este sentido, cabe señalar que el SINBAD *System Classification* se menciona ora como instrumento de evaluación de úlcera en el pie relacionada con la diabetes^(29)^, ora como instrumento de clasificación del potencial de cicatrización^(12)^, e incluso de gravedad de la lesión y predicción de amputación^(16)^.

Por lo tanto, realizar un análisis factorial exploratorio (AFE) y/o confirmatorio (AFC) puede ayudar a verificar la estructura factorial del instrumento e identificar si existen múltiples factores que influyen en el valor de la consistencia interna^(46)^.

A pesar de esta debilidad, este sistema tiene un gran potencial de uso en todo el territorio nacional, especialmente en el contexto de la atención primaria, ya sea para auditoría, como en Inglaterra y Gales, según se citó anteriormente^(49)^, como para justificar la derivación de las personas afectadas a servicios especializados, además de servir para monitorizar eventos que indiquen complicaciones, como la aparición de infección, contribuyendo en ambos casos a prevenir la amputación^(27)^.

Aunque la tasa de respuesta fue baja, considerando el contacto inicial para la validación de contenido, es importante destacar que se cumplió el perfil y el número de expertos establecidos en la literatura^(35-36)^.

La disponibilidad de un sistema adaptado transculturalmente y probado en cuanto a validez de contenido puede contribuir a sustentar la práctica clínica de los profesionales que atienden a personas con úlcera en el pie relacionada con la diabetes. Este sistema posibilita el rastreo de complicaciones, el seguimiento sistematizado y la derivación a servicios especializados, brindando mayor seguridad a las conductas destinadas a esta población. El instrumento puede favorecer la mejora del cuidado y del seguimiento de las úlceras en los pies relacionadas con la diabetes en Brasil, facilitando la comunicación entre los profesionales de la salud y la toma de decisiones clínicas basadas en evidencias.

## Conclusión

El SINBAD *System Classification* está adecuadamente adaptado y validado al portugués de Brasil, con altos índices de concordancia entre los expertos en relación con la pertinencia de los ítems. Esta versión está lista para utilizarse en la práctica clínica y constituye una herramienta válida para la evaluación de úlceras en el pie relacionadas con la diabetes. Sin embargo, es importante resaltar que el sistema aún presenta baja consistencia interna, lo que hace necesario estudiar la validez de constructo del instrumento y la posible incorporación de nuevos ítems.

## Data Availability

Todos los datos generados o analizados durante este estudio están incluidos en este artículo publicado.
